# Ultrafast Near-Field Dynamics in Silver Nanowires Driven by Few-Cycle Short-Wave Infrared Pulses

**DOI:** 10.1021/acsphotonics.6c00044

**Published:** 2026-03-31

**Authors:** Nelia Zaiats, Lukas Wittenbecher, Ivan Sytcevich, Anne-Lise Viotti, Chen Guo, Chandni Babu, Xiaolu Zhuo, Luis M. Liz-Marzán, Eduardo J. C. Dias, F. Javier García de Abajo, Jan Vogelsang, Anne L’Huillier, Cord Arnold, Anders Mikkelsen

**Affiliations:** † NanoLund, 5193 Lund University, Box 118, Lund 221 00, Sweden; ‡ Department of Physics, 5193 Lund University, Box 118, Lund 221 00, Sweden; § Division of Chemical Physics, 5193 Lund University, Box 124, Lund 221 00, Sweden; ∥ CIC biomaGUNE, 90216 Basque Research and Technology Alliance (BRTA), Donostia-San Sebastián 20014, Spain; ⊥ Ikerbasque, Basque Foundation for Science, Bilbao 48009, Spain; # POLIMA-Center for Polariton-driven Light-Matter Interactions, University of Southern Denmark, Odense M 5230, Denmark; ¶ ICFO-Institut de Ciencies Fotoniques, 172281 The Barcelona Institute of Science and Technology, Castelldefels, Barcelona 08860, Spain; ∇ ICREA-Institució Catalana de Recerca i Estudis Avançats, Passeig Lluís Companys 23, Barcelona 08010, Spain; ○ Carl von Ossietzky Universität Oldenburg, Institut für Physik and Center of Interface Science, Oldenburg 26129, Germany

**Keywords:** time-resolved imaging, near-field dynamics, silver nanowires, short-wave infrared pulses, photoemission electron microscopy

## Abstract

Time-resolved imaging of plasmonic near fields is well-established at visible wavelengths but remains largely unexplored in the short-wavelength infrared (SWIR) range. Here, we use time-resolved photoemission electron microscopy (TR-PEEM) to directly visualize ultrafast plasmon dynamics in silver nanowires driven by a few-cycle SWIR pulses. We observe strong electric-field enhancement localized at the nanowire ends, oscillating synchronously with the SWIR optical cycle. Both the local intensity and the temporal response vary within and between individual nanowires. Time-dependent linear modeling reveals that subwavelength variations in nanowire length significantly affect the plasmon dynamics, while the precise end morphology influences both dynamics and field enhancement. Electromagnetic simulations predict additional substrate-induced plasmon modes beyond the nanowire’s fundamental longitudinal resonances. Because the imaged photoemission signal depends highly nonlinearly on the local electric field at the wire–vacuum interface, it is dominated by emission from the nanowire ends, suppressing weaker substrate-induced modes. The photoemission yield shows a lower-than-expected power-law dependence with incident field intensity, suggesting the potential onset of strong-field effects beyond simple perturbative multiphoton emission. These results establish silver nanowires as efficient local SWIR field concentrators and demonstrate that their tunable ultrafast plasmonic responses can be imaged and filtered using photoelectrons, offering promising avenues for nanoscale photonic applications and ultrafast control of electron emission.

## Graphical abstract

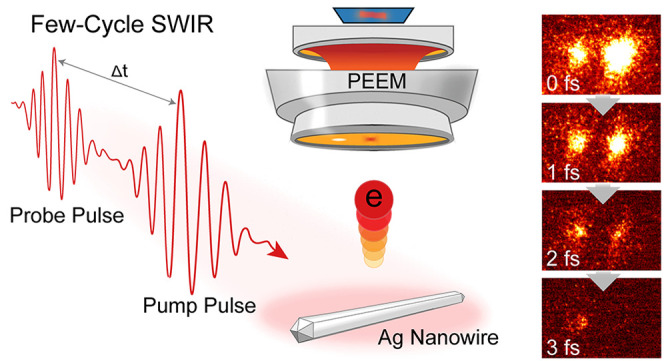

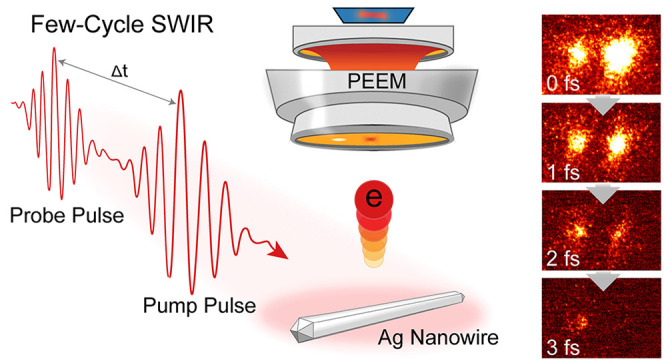

## Introduction

Plasmon-induced local field resonances enable fundamental studies of strong field excitation, the coupling of classical and quantum physics, and ultrafast phenomena,^1–4^ as well as applications including nanoscale lasers, photocatalysis, photovoltaics, and optical information processing.^5–8^ Plasmonic nanoparticles act as strong light concentrators with tunable spectral and polarization sensitivity.^9,10^ In addition, plasmon-driven photoemission enables electron sources with extreme spatial and temporal confinement.^11^ This can be used for imaging and attosecond studies,^12^ for investigations of correlated electron phenomena,^13–15^ and potentially for petahertz computing schemes.^2^

Applications of plasmon excitations have been suggested in a wide range of wavelengths from ultraviolet (UV) to microwave.^16,17^ In the short-wave infrared (SWIR) range (∼1000–3000 nm), plasmonic effects in metal nanoparticles have been investigated for a wide range of purposes. For instance, SWIR wavelengths enable high spatial–temporal resolution in vivo imaging, which can be enhanced through plasmonic nanostructures such as gold nanorods.^18^ Also, SWIR light can propagate through harsh weather environments, making SWIR photodetectors interesting not only for imaging and communication^19^ but also for spectroscopic measurements,^20,21^ thermal imaging, and material characterization.^22^ The performance of photodetectors can be further improved through the integration of plasmonic nanostructures,^23^ and plasmon-assisted enhancement is relevant for advanced optical communication and quantum technologies based on InAs and InSb.^24,25^ Furthermore, SWIR light can drive nonlinear processes such as high-order harmonic generation (HHG), enabling ultrafast studies of plasmonic nanostructures using extreme ultraviolet light with element-specific contrast and attosecond temporal resolution.^26–28^ Most of these SWIR studies employ continuous-wave or nanosecond-to-picosecond excitation, whereas in this work, we use few-cycle (17.5 fs) SWIR pulses to access the ultrafast, subcycle dynamics of the plasmonic near fields.

Silver (Ag) nanowires are well-established for both applications involving plasmonic antennae^29–31^ and fundamental studies of plasmonic near-field dynamics^32,33^ due to their well-defined geometry, high crystallinity, and resonances across a broad spectral range that enable efficient light concentration at the nanoscale.^9,34,35^ In general, Ag nanowires can be synthesized with controlled dimensions tailored to specific applications.^9,36,37^ Ag nanowires predominantly support longitudinal plasmon modes that produce localized hotspots depending on the excitation wavelength and the length of the wire.^5,9,33,38,39^ However, a strongly interacting substrate, such as silicon (Si), can result in the appearance of additional transverse modes (TMs) due to the nanowire–substrate interface interaction.^40^

As plasmon excitations are strongly confined in space and often have very short (5–50 fs) lifetimes,^41,42^ spatiotemporal imaging combining femtosecond (fs) temporal resolution and nanometer (nm) resolution is highly relevant.^43–45^ Time-Resolved PhotoEmission Electron Microscopy (TR-PEEM) is therefore an excellent technique for studies of plasmonic processes, combining the temporal resolution of light pulses with the unparalleled spatial resolution of electron microscopy. It has been successfully applied to reveal near-field dynamics with attosecond precision and to resolve local dark (nonradiative) plasmonic modes.^46–50^ However, direct nanoscale imaging of ultrafast SWIR-driven plasmon dynamics remains largely unexplored. Although most TR-PEEM studies employ pulse durations on the order of tens to a few hundreds of femtoseconds,^47,51^ using shorter pulses spanning a few optical cycles is important for capturing the complex ultrafast dynamics characteristic of plasmonic systems that can decay within just a few cycles.^32,52^

Here, we use TR-PEEM to image nanoscale photoemission from Ag nanowires excited by two 17.5 fs SWIR pulses separated by a variable temporal delay. This allows the capture of the femtosecond evolution of plasmons driven by SWIR excitation. Delay-dependent TR-PEEM measurements reveal oscillating near-field hotspots at the nanowire ends with distinct amplitudes and temporal phases. Time-dependent modeling reproduces the measured spatiotemporal field distributions, indicating that nanowire length primarily determines the temporal dynamics, while tip morphology controls both the dynamics and the field enhancement. Power-dependent measurements indicate effective photon orders of 3–5, below the expectation of 6–7 from perturbative multiphoton excitation. This deviation could indicate the onset of strong-field contributions, while local variations in surface chemistry may also influence the emission scaling. Our results show that Ag nanowires can act as SWIR local field concentrators, capable of driving field-controlled photoelectron emission on a few-femtosecond time scale.

## Results and Discussion

### Experiment

The experiment is sketched in Figure 1a. Two SWIR pulses with identical polarization and carrier-envelope phase (CEP) are directed toward the sample in the PEEM at an angle of 65° with respect to normal incidence. The time delay between the SWIR pulses can be varied by using an interferometer. The released photoelectrons are imaged in the PEEM to create a magnified image with a resolution down to ∼50 nm. The spectral and temporal profiles of the SWIR pulses span a 1700–2400 nm spectral range (Figure 1b) as a broad spectrum is required to create pulses with a 17.5 fs fwhm temporal width (corresponding to 2–3 cycles of the optical field). The experimental setup used to produce these pulses is described in ref 53 and in the Methods section. The pulse energy reaches up to 13 μJ before the interferometer and is attenuated to avoid space–charge effects. Relative intensity variations are monitored, and the linear polarization can be systematically adjusted.

**Figure 1:**
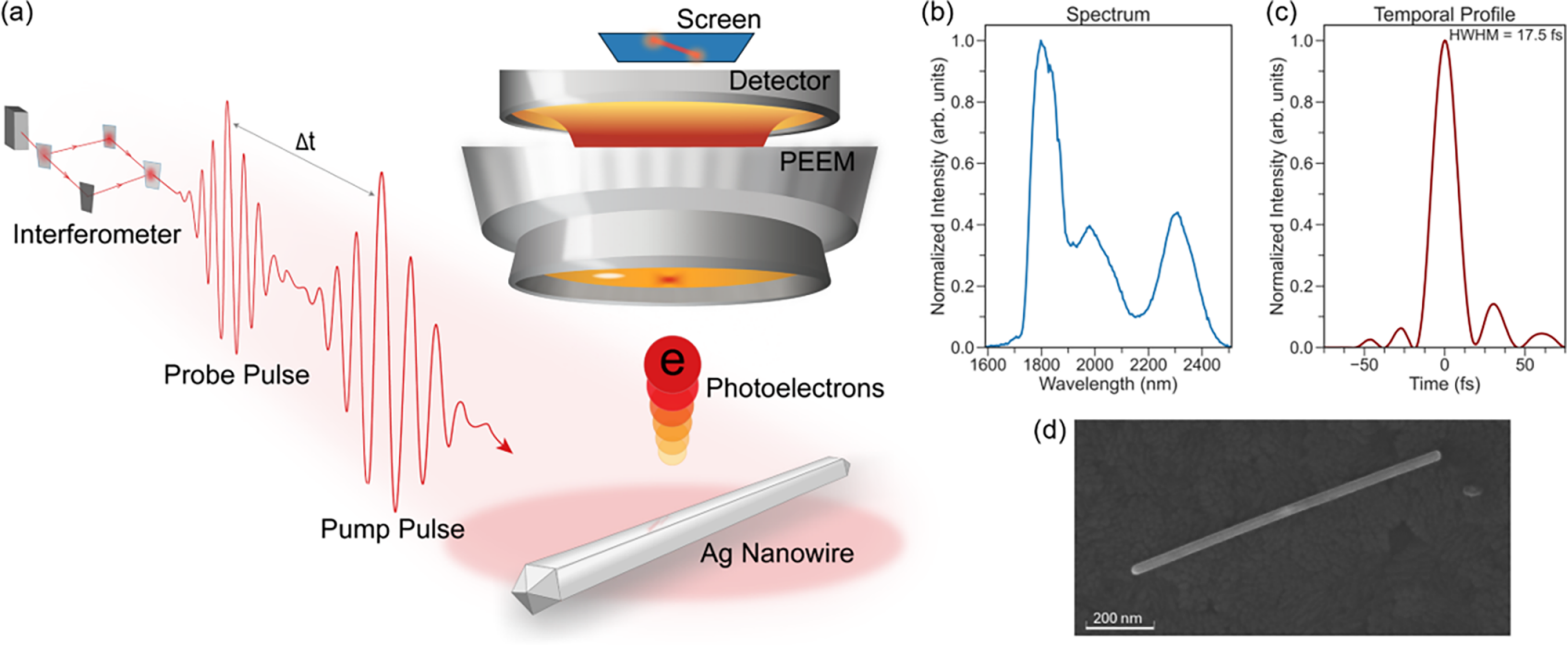
Experimental setup and laser characterization. (a) Schematic of the experimental setup for TR-PEEM measurements on a silver nanowire. A pump–probe scheme is used, where an interferometer controls the delay (Δ*t*) between the pulses. The interaction of the pump and probe pulses with the nanowire generates localized near-field enhancements at the wire, leading to the emission of photoelectrons, which are then imaged using PEEM. (b) Spectral characteristics of the laser pulse. The laser spectrum spans from 1600 to 2500 nm. (c) Temporal profile of the laser (retrieved as described in the Methods section) with a full-width at half-maximum (fwhm) of 17.5 fs. (d) Scanning electron microscopy (SEM) image of a silver nanowire as deposited.

We studied Ag nanowires with a well-defined elongated geometry and crystal structure (Figure 1d). They had lengths of 900 to 1100 nm and widths of ∼32 nm. The nanowires are synthesized from a small bipyramid Au core (visible as a brighter dot in the middle of the nanowire in Figure 1d) that is coated by ∼1 nm Ag.^33^ This ensured well-defined crystallinity, high aspect ratios, and uniform morphology.^33^ At wavelengths >1000 nm, Au and Ag should exhibit similar plasmonic responses.^33^ Further, since the surface is Ag-coated, we treat the nanowires as effectively pure Ag in the following. The symmetric shape of the nanowires promotes a simple distribution of the (longitudinal) plasmon modes.^44,54^ The synthesis involved the cetyltrimethylammonium bromide (CTAB) surfactant, which prevents aggregation and can remain as a 1–2 nm surface coating after deposition.^55^ This organic layer has only a minor influence on the plasmonic response in the studied spectral range.^56^ Nanowires were placed on a standard Si wafer with a thin ∼3 nm SiO_2_ native oxide. The nanowires were randomly dispersed, and thus, wires with specific orientations could be studied by imaging different areas of the sample using PEEM.

### Local Field Shape and Dynamics

Photoelectron imaging using a mercury (Hg) arc UV source for excitation (Figure 2a left) was used first to visualize the nanowire placement and orientation on the substrate. Figure 2a shows two distinct nanowires with lengths of ∼1000 nm. When illuminated with SWIR pulses, photoelectrons are emitted primarily from the nanowire ends, as observed in the right image of Figure 2a. The bright emission spots are due to field enhancement and can be interpreted as a consequence of excitation of longitudinal SPP cavity (Fabry–Pérot-type) modes of the finite-length nanowire, which generates strong near-field maxima at the terminations. The spatial field distribution will therefore depend on the nanowire geometry, while the degree of mode excitation depends on the driving wavelengths.^33,34,57^

**Figure 2:**
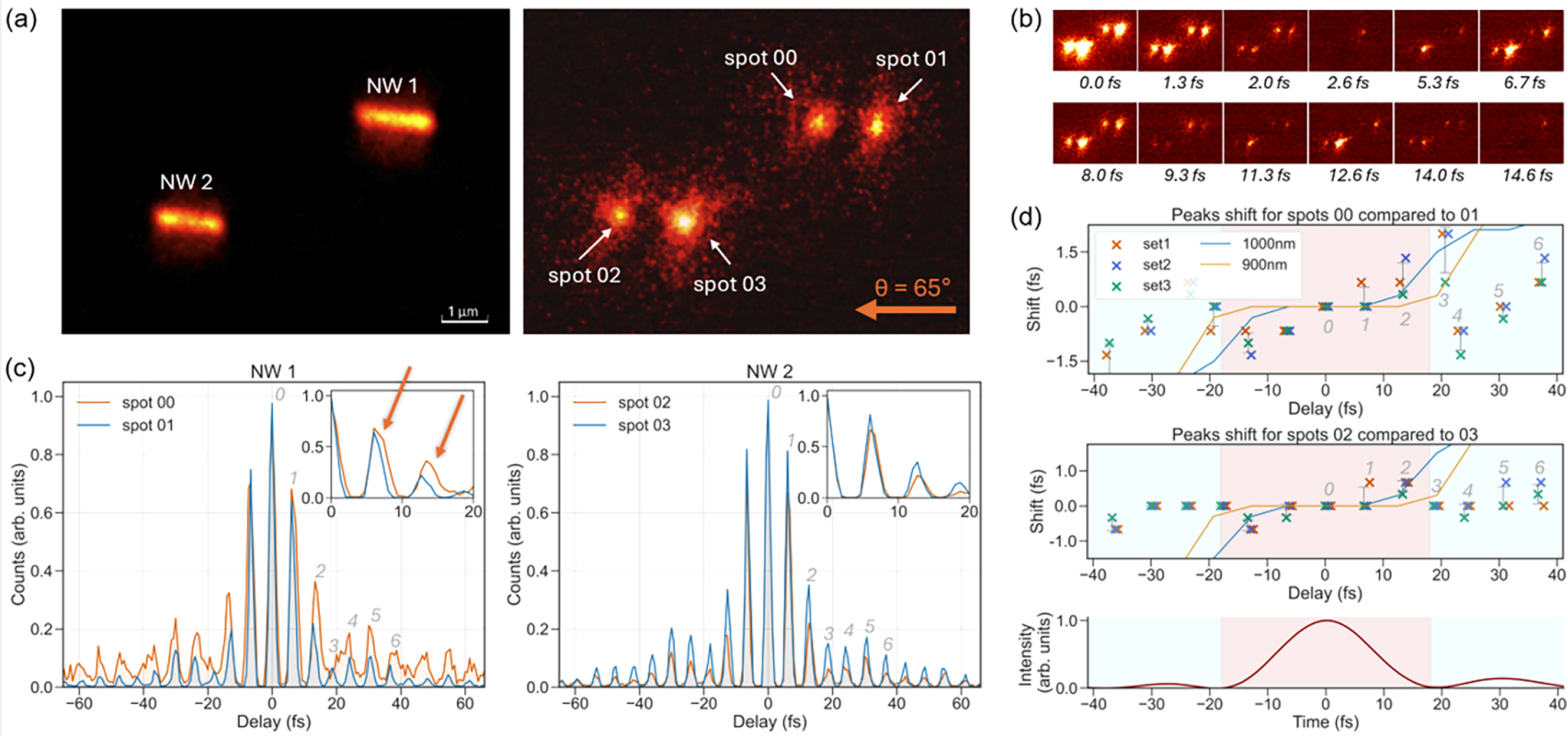
Near-field dynamics in Ag nanowires studied using TR-PEEM. (a) PEEM images of two Ag nanowires (NW 1 and NW 2) excited using a Hg lamp (left) and the SWIR laser source (right). The laser-excited image reveals distinct localized bright emission spots (00, 01, 02, and 03), corresponding to elongated and asymmetric field enhancements at the ends of the nanowires, arising from localized plasmon resonances. The direction of light pulses on the sample is indicated by the orange arrow, which is at a 65 degree angle from the surface normal. (b) The time-dependent PEEM images recorded with the laser source show the evolution of localized plasmon resonance at different time delays between excitation pulses. The images shown here are representative frames; the full delay scans were acquired with a temporal step size of 0.67 fs. (c) Interferometric autocorrelation (IAC) traces recorded at selected emission spots (00 and 01 for NW 1, 02 and 03 for NW 2). Each peak in the trace corresponds to an interference between pump and probe pulses, with time “0” defined by the maximum of the local IAC of each spot, indicating ultrafast plasmonic dynamics. Insets highlight zoomed-in peak shifts between different spots happening during the first 20 fs delay times. Peak positions are extracted as described in the Supporting Information. (d) Extracted peak shifts from the IAC traces for NW 1 (top) and NW2 (bottom), comparing pairs of emission spots (00 and 01 for NW 1, 02 and 03 for NW 2) for three separate data sets and revealing spatial variations in plasmonic response. Thin solid curves represent peak shifts for two different simulated lengths of the nanowire (1000 and 900 nm) to indicate possible differences due to length. The bottom panel shows the temporal profile of the excitation pulse, highlighting the main interaction region (red) within ±18 fs and the side-lobe pulse regions (blue) outside this window.

At SWIR wavelengths, these modes have a combined propagating SPP character with local field enhancement at the ends, for example, lowest-order antenna-like modes. This results in the appearance of end hotspots in PEEM images. The central part of the nanowires shows little to no emission, which is consistent with the interpretation in terms of plasmon resonances and the high nonlinearity of the photoemission, as discussed below. Several polarization scans of hotspots in individual nanowires were performed, as shown in the Supporting Information. A dipole-like intensity dependence on the polarization angle was observed, with a maximum when the polarization vector projection on the surface was parallel to the nanowire’s long axis. This is consistent with plasmonic enhancement. For the experiments shown in Figure 2 we used p-polarization, which gave the strongest emission for the nanowires.

To measure the temporal evolution of the local fields, we vary the time delay between the two SWIR pulses and record a sequence of TR-PEEM images, as exemplified in Figure 2b. These images show the modulation of photoemission intensity at the ends of two different nanowires, with localized hotspots appearing, disappearing, and reappearing periodically. This periodic modulation approximately follows the optical carrier cycle (∼7 fs) of the SWIR pulse, giving space- and time-resolved information on the electron emission process. Comparing the emission from the hotspots, the photoemission intensities are not identical and vary both from wire to wire and locally within a single nanowire. As the orientation of the two nanowires in Figure 2 differs by only 7–8° (their long axes are nearly aligned with the illumination direction) and they are located within 3 μ m of each other, the differences are likely due to the geometric end shape or the surface variations between the wires and not their geometric alignment with respect to the beam. This is corroborated by the theoretical simulations (see below).

To probe the temporal evolution of the localized fields in more detail, we integrated the photoemission signal from each of the bright ends of the nanowires in the images and plotted the obtained yield as a function of the delay between the SWIR pulses (Figure 2c). To interpret these results, we use the fact that the interferometric autocorrelation (IAC) traces of the combined excitation of the two SWIR pulses can (in a basic multiphoton photoemission picture) be calculated based on the superposition principle for electric fields and the order of the multiphoton process^32,58^

1 $$I\left(r,\Delta t\right)=\int {\left|E_{loc,pulse1}\left(r,t\right)+E_{loc,pulse2}\left(r,t+\Delta t\right)\right|}^{2n}dt$$

*I*(*r*, Δ*t*) is the position and time-dependent photoemission signal, *E*_loc,pulsei_ is the local electric field induced by the pulse *i*(*i* = 1, 2) at position *r* on the surface, and *n* is the number of photons needed to release a photoelectron into the vacuum. In a perturbative model, *n* will be equal to the number of photons needed to overcome the work function barrier of the Ag wires (between 6 and 7, see below). The resulting photoemission signal is thus a highly nonlinear function of the local field intensity at the nanowire surface, which makes it sensitive to very small variations in the field amplitude, allowing us to trace the detailed time-resolved dynamics of the local near-field response. Further, any regions with minor field enhancement will not be visible because very few electrons are emitted (e.g., a doubling in field strength will lead to a 1000-fold emission increase already for 5-photon photoemission). We note that factors such as the work function and excitation probabilities due to the band structure can also affect the absolute intensity of the photoemission.^59,60^

The measured IAC traces (Figure 2c) reveal sharp interference peaks, with the strongest peak at 0 fs delay, corresponding to the two pulses overlapping precisely in time. The periodicity of the traces (∼7 fs) is approximately half of the SWIR pulse electric field, as expected for an interferometric autocorrelation measurement, which has maxima at constructive interferences between the two pulses. The original pulses have both a central envelope (fwhm 17.5 fs) and side lobes (see Figure 1c) that can also be observed in the IAC traces. However, the precise interference peak intensity and temporal position vary between the nanowire hotspots. By comparing traces from the two nanowire-end hotspots 00–01 (NW 1) and 02–03 (NW 2), as in (Figure 2c), we observe shifts as well as broadening of the peaks. Sometimes, more complex peak shapes, such as double peak structures in peaks 3–4 for spot 00, are observed. The double peak features could be confirmed by the appearance in all three independent trace measurements. The IAC trace changes of individual wires vary (e.g., more significant differences for NW 1 than for NW 2). Again, as the excitation fields at the two wires are identical, these differences in dynamics must be controlled by the specific orientation, shape, and surface of the nanowires.

To quantify the shifts of the interference peaks observed in the IAC traces, we determined the peak positions by fitting the local IAC peak profiles (details in the Supporting Information). The extracted peak shifts are plotted in Figure 2d. The error bars represent the standard deviation of the peak shifts obtained from three independent measurements of the same nanowires with an average value of 0.3 fs. We first focus on the dynamics of delays up to ∼20 fs, corresponding to the large central envelope of the laser pulse. As observed in the insets of Figure 2c and quantified in Figure 2d, an increasing shift between the two hotspots at the wire ends is found for peaks at ∼6 and ∼12 fs, with a maximum shift of ∼1 fs for NW 1 and ∼0.5 fs for NW 2. The peak intensity decays are also different for the hotspots at the two ends, as observed in the insets of Figure 2c. The shifts are reproducible in three separate time series experiments (Figure 2d) on the same wires, even if there is minor variation between the three traces. These variations can be explained by small changes in the spectral profile of the pulse over time that can favor excitation of different plasmon modes in the nanowires, with different resonance wavelengths (as discussed in more detail below and seen in the simulation of the excitation spectra in Supporting Information Figure S10) or slight changes in the pulse CEP. Qualitatively, since the light pulses are only 2–3 optical cycles long, they inherently contain a broad frequency spectrum, and different frequency components couple differently to the available plasmon modes. This frequency mismatch leads to a temporal dephasing between the driving field and the induced plasmonic oscillations, which results in temporal shifts between the hotspots. Although the excitation field is identical at both wire ends, the plasmonic response differs locally, leading to a different temporal phase evolution of the plasmonic response and thus to relative shifts between the two hotspots belonging to the same nanowire. The observation of the plasmonic transient response is not entirely trivial as the plasmon lifetimes are short (down to 5–10 fs). Their influence on the measured IAC traces will be small if they are much shorter-lived than the light pulse duration. This plasmonic dynamics can explain the difference in the degree of response of the two nanowires in Figure 2. If the plasmon lifetime is shorter in NW 2 than in NW 1, the hotspot oscillations follow more closely the driving field with less phase variation. Geometric differences, such as lengths and end shapes, can lead to altered lifetimes. Differences in lifetimes can also occur due to chemical variations in the surface coating of the wires.^61,62^

Focusing now on longer times from ∼20 to ∼50 fs, the exciting SWIR pulses have nontrivial temporal envelopes with two side envelopes (lobes) beyond the central envelope (see Figure 1b). At early delays, the dominant contribution to the IAC signal comes from the central envelope of the pulse (IAC peaks 0–5, peak 0 being at 0 fs). However, as the pump–probe delay increases, it reaches time scales at which the side envelopes of the SWIR pulses contribute. When the weaker side envelope of the laser pulse overlaps with a central envelope part at a delay from 20 to 30 fs, the IAC shift becomes 0 again. From IAC peaks 4–6 in the top part of Figure 2d, the difference in oscillation frequency is first negative, then 0, and then positive, essentially repeating the pattern of the central peak at 0 fs delay. This behavior can be explained from Figure 2c in that we are initially seeing the plasmon excited by the first central part of the pulse, which is decaying, and a remnant of this is still seen as a shoulder at ∼20 fs in spot 00. However, at this point, the side-lobes of the first exciting SWIR pulse will generate a new plasmon, which will be in phase with the central part of the second SWIR pulse. This effectively reproduces the autocorrelation of the central pulse envelopes, now involving interaction between a side envelope and a central envelope, leading to a repetition of the phase shifts observed between 0 and 20 fs in the 20 and 40 fs range. These effects are quantized in Figure 2d, where the upper graph shows the relative time shift between hotspots 00 and 01 (NW 1), while the lower graph does the same for spots 02 and 03 (NW 2) for three different data sets.

In summary, the photoemitted electron signal from the Ag nanowires is strongly enhanced by the plasmonic response to the SWIR pulses, with the exact enhancement varying from the end of each wire. The photoelectron intensity variation in time reflects the oscillating field of the SWIR pulses with a subfemtosecond difference due to the specific excited plasmonic modes, which vary from wire to wire.

### Modeling of Plasmon-Induced Field Dynamics

To better understand the experimental observations, modeling of the time-dependent electromagnetic field was performed using the finite-difference time-domain (FDTD) simulation method.^32,63^ We modeled a SWIR pulse matching the central temporal structure and spectral bandwidth of the experiment, interacting with a Ag nanowire of comparable geometry on a Si substrate with a 3 nm native oxide. More details on the simulations can be found in the Methods section and the Supporting Information. In Figure 3a ,we show the top and side views of the absolute electric field strength of a 1000 nm nanowire with rounded ends. We also show plots of the calculated IAC traces from the two ends of this wire in Figure 3b. In the Supporting Information (Figure S10), we show the nanowire field enhancement as a function of wavelength as well as field distributions for other wire lengths and corresponding IAC traces. Generally, field maxima are observed at the nanowire ends, with less intense peaks along the wire. Our results can be compared to previous simulations^33^ of the Ag nanowire optical response at similar wavelengths. While the field enhancement found at the ends of the wire agrees with the previous modeling work,^33^ the additional near-field maxima along the central wire were not reported. This can be understood as a modification of the plasmon modes due to the presence of the Si substrate, which has been found at shorter (visible) wavelengths.^40,64^ The presence of the Si substrate alters the resonance wavelengths of the ubiquitous longitudinal plasmon modes,^64^ and new transversal modes (TMs) are also introduced due to the interaction between the substrate and the nanowire.^40^ We confirm this simulation of a weaker interacting (ITO) substrate (see the Supporting Information), which shows near-field distribution patterns that more closely resemble those for substrate-free wires,^33^ although the substrate still affects the longitudinal plasmon resonance, as also found previously for shorter wavelengths.^65^ Simulations and PEEM imaging of Ag nanowires on a Si substrate previously performed for shorter (visible) wavelengths^40^ show the new TMs in the wire and strong enhancement at the interface between Ag and Si. Similar substrate-coupled fields can also be observed in the side-view image in Figure 3a for the SWIR wavelengths. However, due to the highly nonlinear photoemission process for SWIR (power of 6–12 of the electric field intensity), the areas of strongest enhancement (at the ends of the wires) will emit photoelectrons at much higher intensity than the weaker fields in the middle of the wire. At shorter wavelengths (higher photon energies), the emission order will be 4–6 with the field intensity, making weaker fields visible. The strong field enhancement observed at the interface between the nanowire and the Si substrate will excite many high energy photoelectrons in this region, but they will not escape into the vacuum due to the low inelastic mean free path of electrons in matter that will quench any photoexcited electrons from this interface as they pass through the nanowire. Both the dynamics and top-view field shapes (see Supporting Information Figures S11 and S14) of the ends appear qualitatively similar for the two substrates; the difference in photoemission is expressed in the detailed dynamics in the IAC traces at SWIR wavelengths.

**Figure 3:**
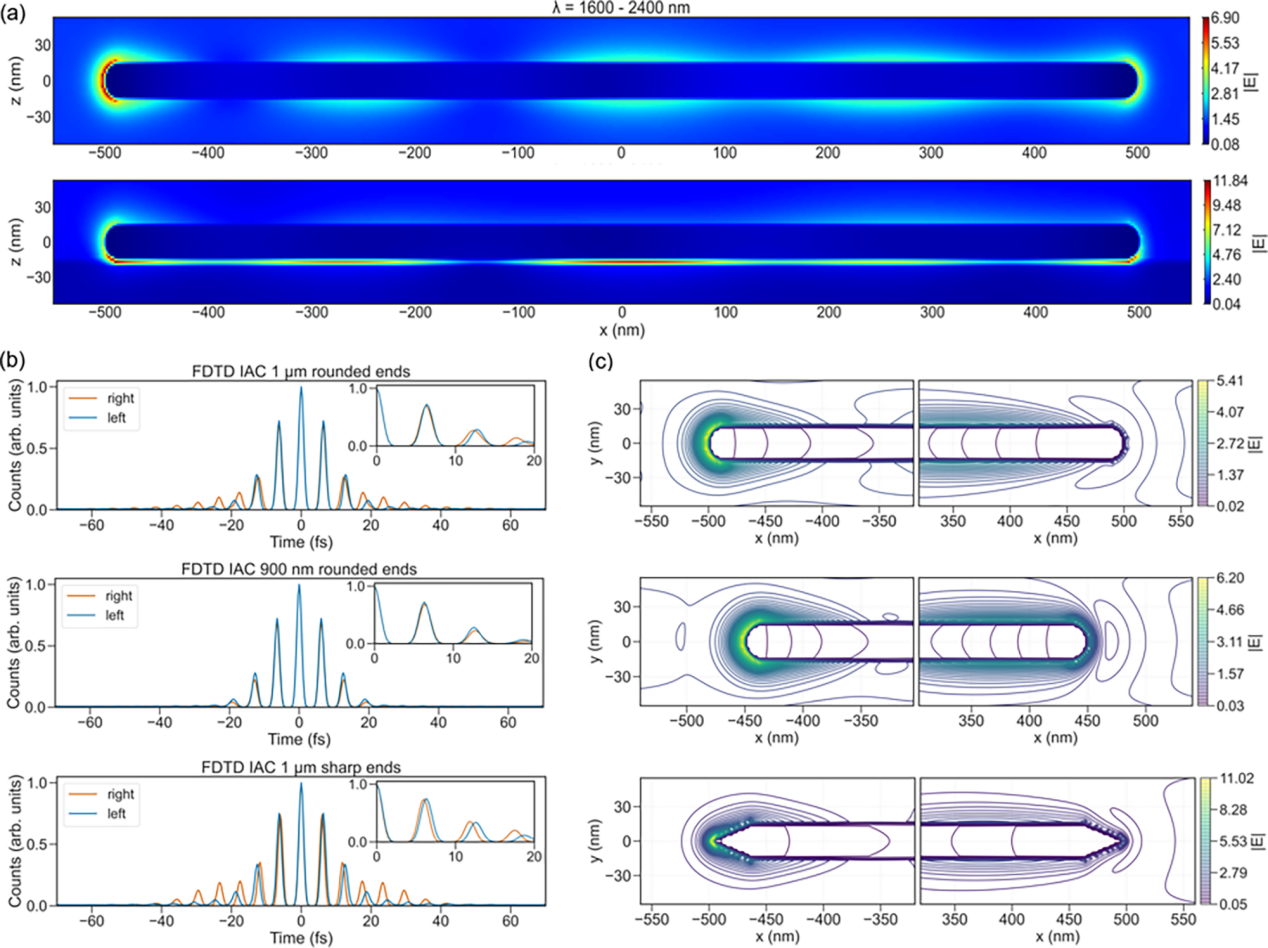
FDTD simulations of electromagnetic fields on Ag nanowires on a Si/SiO_2_ substrate under SWIR light excitation (incident from the right at 65° to the surface normal). (a) Top-view (top) of field distribution |*E*| averaged over the full spectral excitation range (1600–2500 nm) for a 1000 nm nanowire, showing strong field localization at the wire ends and weaker intensity along the wire body. The side-view (bottom) reveals coupling of the plasmonic field with the substrate. (b) Simulated interferometric autocorrelation traces (IAC) at the left and right ends of nanowires of different lengths (1000 nm in the top and 900 nm in the bottom). Insets show the 0–20 fs delay time regions, highlighting differences in symmetry. (c) Top-view equipotential contours at the opposite ends of 1000 nm (top panel) and 900 nm (bottom panel) long nanowires with rounded tips at the central wavelength. (d) Same as (c) for a 1000 nm nanowire with sharp tips, revealing stronger and more confined field enhancement at the ends.

The simulated IAC traces from the two ends of the 1000 nm long nanowire, as observed at the top of Figure 3b, exhibit a difference in oscillation frequency that resembles the observed experimental trend (Figure 2d). For the first few optical cycles, the fields gradually shift out of phase, as observed in experiments. These predicted phase shifts correspond well, even quantitatively, to those experimentally observed in the first three optical cycles (Figure 2d). The quantitative differences in the phase shifts between the two wires can then be explained by the theoretical calculations as small (subwavelength) differences in the wire lengths (e.g., from 1000 to 900 nm in the middle Figure 3b) lead to changes in the magnitude of the phase shifts. The simulations further show that the IAC peak decay depends on wire length, with slower decay for the 1000 nm nanowire than for the 900 nm one. This is consistent with experimental observation, where NW 2 does not show a double peak structure, whereas NW 1 does (Figure 2c).

Instead of variation of the length, the shape of the nanowire ends can also be changed. In Figure 3b (bottom panel), we show the IAC traces from a 1000 nm wire with sharp ends. Modifying the end shape increases the phase-shift difference between the two ends and slows the decay of the IAC peak height. Changes in the length or shape of the nanowires at the scale of ∼100 nm (about 1 order of magnitude less than the SWIR wavelength) can be used to control the dynamics of the SWIR-induced plasmon enhancement and subsequent photoemission from the nanowires.

The broadening and more complex peak structure in the experimental IAC traces at longer delays (>15 fs, Figure 2c) are not observed in the theoretical modeling of Figure 3. Such behavior can be found for wires with differently shaped ends placed on Si, as seen in Figure S13. However, in the present case, this can also be attributed to the more complex temporal structure of the experimental pulse compared to the pulse used for modeling (which does not have side lobes, only the central envelope). The central SWIR peak excites longitudinal plasmon modes that oscillate with a phase delay relative to the SWIR pulse, and consequently, as the side peak pulses appear and if the excited plasmon modes have not already decayed, they will mix, leading to a double peak structure in the IAC oscillations, visible in Figure 2c peaks 3–4 of spot 00. The visibility of this feature depends on signal-to-noise ratio and the effective plasmon lifetime.

The equipotential line plots in Figure 3c,d visualize the near-field distribution around nanowires of different geometries and highlight how the field symmetry depends on wire length. It can be seen that while the length influences the IAC trace dynamics, it has little influence on the field intensity. However, changing shapes strongly influences the maximum intensity of the fields, as seen by comparing Figure 3c and d. As expected, sharper nanowire tips significantly enhance the local field compared to rounded ends. However, the exact shape of the fields also differs with the rounded shape, resulting in a sickle-shaped field distribution, as indicated by the field lines in Figure 3c, while sharper ends exhibit a more isotropic field distribution.

As we observe a photoemission signal proportional to the field strength and the power of 6–10, even small changes in tip geometry can strongly amplify the PEEM signal. While variations in the strength of the fields at the surface of the nanowire, where the excited electrons originate, lead to substantial changes in the observed photoemission intensity, the spatial shape of the electric field affects the spatial distribution of the electron emission. The emitted electrons have energies of a few eV when they leave the surface and pass through the local plasmon-enhanced fields, where their trajectories are distorted by the field lines.^66^ As a result, the spatial field distribution, as shown in the theoretical calculations, should be reflected in the real space distribution of the emitted electrons in the PEEM image. Thus, the ends can be used to manipulate the intensity and geometric distribution of the electric fields, while the nanowire length mainly governs the temporal dynamics. A more detailed comparison of electric fields and reproducibility of the measurements and simulations is also described in the Supporting Information Figures S9–S12.

### The Nonlinear Photoemission Process

To monitor the induced field strengths of the plasmon hotspots, we measured their intensity as a function of laser power (Figure 4). While the absolute on-sample intensity can only be estimated, the relative change can be measured using a power meter before the SWIR beam enters the PEEM vacuum chamber. The power dependence of the photoemission process was estimated from log–log plots of the photoemission yield as a function of excitation power (Figure 4a) by fitting the data with a power-law behavior (as in eq 1). The power-law fits are performed over the experimentally accessible relative laser power range from 40 to 90, corresponding to a photoemission yield change spanning a factor of ∼40. The limits were set by the lack of a signal at low laser powers and the onset of a space charge effect at high laser powers. While this range is enough to determine the power-law exponent, it is too limited to reliably observe transitions from the photoemission mechanism to another that would require fitting of two different exponents.^3^

**Figure 4:**
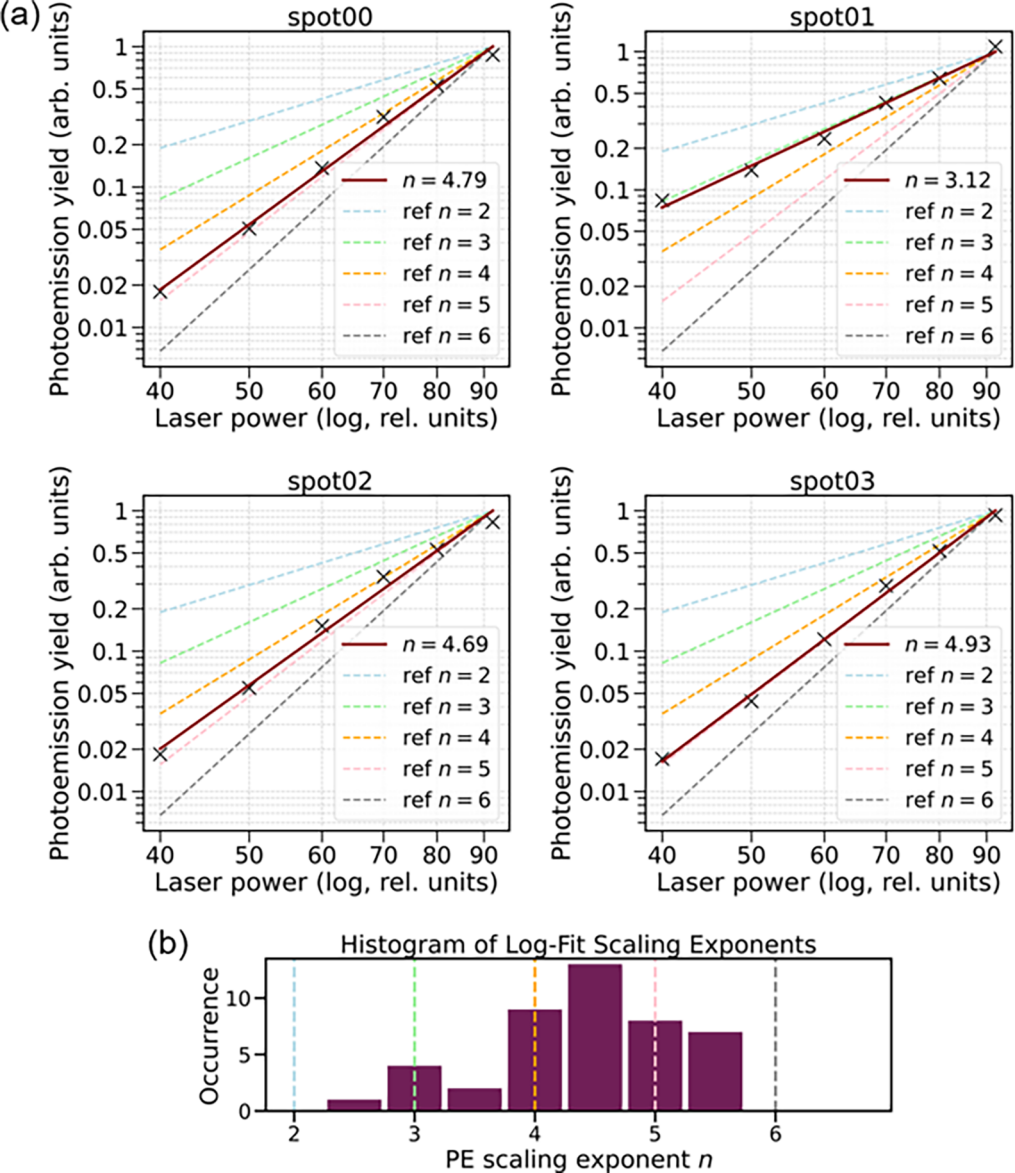
Intensity dependence of the total photoemission signal from nanowire ends. (a) Power-law scaling curves of the photoemission yield as a function of laser power for four nanowire emission spots. Log–log fits are shown with the extracted exponents indicated. Scaling exponents for *n* = 2–6 are marked with dashed lines. The plotted yield is normalized to maximum observed yield for each spot. (b) Histogram of scaling exponents obtained from log–log fits across all measured data sets. The dominant nonlinearity is found between 4 and 5 photons. The expected six-photon scaling is marked with a gray dashed line.

In Figure 4b, we show a histogram of the exponents of measured power laws for 20 different nanowires and their hotspots. The extracted exponents range from 3 to 5.5, with the majority at 4–5. In a basic nonlinear perturbative multiphoton emission picture, these numbers correspond to the combined number of photons needed for an electron to gain sufficient energy to overcome the work function barrier. The work function of Ag nanowires can vary due to the surface geometry or the presence of adsorbed molecules.^67^ Adsorption of organic molecules (including bromide-containing organic molecules such as the used CTAB surfactant) has resulted in work functions between 5.2 and 3.7 eV.^68–70^ The highest photon energy of our SWIR pulses (with significant intensity) is 0.7 eV. This implies that at least 6 photons are required to emit photoelectrons over a threshold of 3.7 eV, which disagrees with the observed values of 3–5.5. For 3–4 photon emission, the work function would need to be <2.8 eV. Lowering the work function to such values is possible using a submonolayer adsorption of pure metallic alkalies;^71,72^ however, this is not a situation found in the present samples. It can be noted that for a range of other studies using shorter wavelength optical pulses, simple multiphoton photoemission has been found^32,46,49,73^ as the intensity needed to overcome the work function threshold is smaller.

From these considerations, we conclude that the possible values of the work function cannot explain the low power-law exponent, and instead, other emission mechanisms must contribute. Two such mechanisms are optical tunneling (field emission) and thermionic emission.^74,75^ The latter is a result of the broadened electron kinetic energy distribution that occurs at high intensity excitation of free electrons in the metal as the effective electron temperature is increased by depositing optical energy in the electronic system. If sufficiently thermalized, electrons with a high energy could be more easily excited by the field, thus resulting in a lower number of photons needed for photoelectron emission. Given the 200 kHz repetition rate (5 μs pulse separation), the electrons have time to cool between pulses. To assess whether heating could still reduce the effective nonlinearity in a single shot, we calculated the temperature evolution using the simulated near fields (see the hot-electron-temperature dynamics section in the Support Information). Because the near-field distribution is nonuniform across the nanowire (e.g., concentrating at the ends, as discussed), the temperature is likewise nonuniform initially, until heat diffusion (acting on a picosecond time scale) eventually homogenizes it. *T*_max_ always occurs at the surface and is the strongest at the wire ends, so it is the most relevant quantity for PEEM. For an incident fluence of *F*_0_ ≈ 18 mJ/cm^2^, the local electron temperature rapidly rises above 5000 K on a femtosecond time scale before cooling slowly (via diffusion and electron–phonon coupling), reaching a uniform state after approximately 8 ps and returning to ambient temperature after more than 10 ps. At this fluence, the chemical-potential shift is only 0.01 eV (Figure S15), which is insufficient to significantly reduce the work function. For comparison, even at a fluence ten times higher than the one used in this work, the estimated shift would remain too small to account for the observed scaling.

Another possibility is that we have reached the optical tunneling regime in which the work function barrier is distorted by the field, allowing tunneling with a substantially smaller effective number of photons. This high field regime^74,76–79^ can become relevant at the high intensities of the few-cycle SWIR pulses. In particular, in the presence of plasmonic field enhancement, such transitions have been observed.^80^ To evaluate this mechanism, we estimate the field intensity at the Ag nanowires and compute the corresponding Keldysh parameter, defined as $\gamma =\omega \sqrt{2m_{e}\Phi }/eE$ , where ω is the angular frequency of the incident light, *m*_e_ is the electron mass, Φ is the local work function of Ag, *e* is the elementary charge, and *E* is the local electric field amplitude at the nanowire surface. The peak electric field *E* at the sample is obtained from the estimated pulse energy, measured spot size, and pulse duration, assuming Gaussian temporal and spatial profiles. Using a single-pulse energy of *E*_pulse_ ≈ 2.4 μJ, a pulse duration of 17.5 fs, and an elliptical focal spot of ∼201 × 85 μm^2^, we obtain a peak intensity of *I*_max_ = 6.6 GW/cm^2^. This corresponds to a peak electric field of *E*_0_ ≈ 2.2 × 10^9^ V/m. Considering a local near-field enhancement factor of 5–10 at the Ag nanowires, a work function of Φ = 4.6 eV for silver, and an excitation wavelength in the range 1.7–2.4 μm, the resulting Keldysh parameter is γ ≈ 0.1–1. This value indicates the approximate excitation mechanism: γ ≫ 1 corresponds to the weak-field multiphoton regime, while γ ≪ 1 signifies the strong-field optical tunneling regime. The intermediate values obtained here place the emission in the mixed (or transition) regime, where both multiphoton and tunneling contributions can coexist and play a role in the photoemission process.^76^ More detailed calculations can be found in the Supporting Information. From the power-law dependence on the excitation intensity, we would conclude that we are in a regime where optical tunneling combined with multiphoton excitation is possible. The variation in power-law behavior observed for different nanowire hotspots can also be understood in this regime. Here, the phase and the detailed field distribution are important, and thus, the exact shape of the plasmonic nanostructure can modify the local field enhancement and hence the power-law dependence.^74,76–79,81^

## Conclusion

Photoemission from Ag nanowires excited by few-cycle SWIR pulses has been studied using TR-PEEM. We observe photoemission hotspots at the ends of the nanowires with an intensity reflecting the optical oscillations of the SWIR pulses with additional structure and phase shifts produced by the plasmonic response. While theoretical modeling shows a more complex field distribution across the nanowire, high-order nonlinear photoemission reveals dominant photoelectron hotspots at the nanowire ends. Theoretical modeling further indicates that by varying the length and the end morphology of the nanowires in the 100 nm range (far below the wavelength), the temporal and spatial distribution as well as the intensity of the photoemission signal can be controlled. The introduction of a strongly interacting Si substrate adds additional plasmon modes (due to splitting in out-of-plane and in-plane transverse plasmons of the nanowire) and complicates the field distribution. However, the effect on photoemitted electrons is limited, although an enhancement of the excited electrons inside the nanowire can be expected. While most previous photoemission experiments are performed with photons sufficiently energetic to give 1st to 3rd order photon photoemission, the use of SWIR photons results in emission only at orders 3 and upward. This generally leads to a stronger field sensitivity and dominant emission from the strongest field regions. Comparing to studies at shorter wavelengths,^40^ this can lead to a simplified emission in the present case, as emission except from the ends of the nanowires is filtered out. Thus, SWIR pulse excitation of an ensemble of nanowires on Si would presumably lead to simpler emission dynamics, less influenced by individual wire behaviors. Further, as studies of optical fields using PEEM are space–charge-limited, the increase in field intensity before significant emission occurs can open up for spatially resolved studies of high field emission. While our studies indicate that we are beyond the basic multiphoton emission regime, future studies on the photoemission processes due to SWIR pulses would be interesting to fully understand this phenomenon. The present measurements were performed in a conventional PEEM instrument without energy resolution of the emitted electrons. Using a PEEM setup equipped with an electron energy analyzer, such as time-of-flight,^82^ would allow a detailed study of the photoelectron energy distribution and a simultaneous quantitative work function determination. Our findings open new opportunities for the use of SWIR-driven plasmonic nanostructures in nanophotonics experiments and demonstrated that few-cycle SWIR pulses combined with PEEM provide a powerful platform for mapping plasmon dynamics with nanometer and femtosecond precision. With the simpler emission patterns observed from SWIR-excited nanostructures, the temporal profile of electrons photoemitted from an oriented ensemble of nanowires excited in this wavelength regime may also be clearer and, hence, more useful.

## Methods

### TR-PEEM Setup

The TR-PEEM setup (from Focus GmbH) was operated under ultrahigh vacuum conditions (base pressure ∼1 × 10^–9^ mbar). A mercury lamp was used for continuous UV illumination, alignment, and reference imaging. For time-resolved pump–probe experiments, femtosecond laser pulses were directed onto the sample surface under an incidence angle of 65° with respect to the surface normal, creating an elongated spot of 201 × 85 μm and ensuring homogeneous excitation across the PEEM field-of-view. The 65° incidence can introduce a geometric delay along a 1 μm nanowire of up to ∼3 fs, but since pump and probe pulses arrive from the same direction, this produces only a constant offset in the time zero that is accounted for and does not affect the relative pump–probe delay between emission spots.

The photoelectrons generated at the sample surface were accelerated in a strong extraction field and guided through the PEEM column, where they were imaged onto a channel plate-based detector. This allows direct mapping of local variations in photoemission yield with spatial resolution down to tens of nanometers depending on illumination conditions and the accelerating voltage in the PEEM column. By recording the static Hg lamp imaging and comparing them with the pulsed laser excitation, the setup allowed us to directly compare the photoemission due to field oscillations and ultrafast optical excitation with the geometric alignment of the nanowire.

### Excitation Pulses

To characterize the temporal profile of the SWIR pulses, we used the third-harmonic generation (THG) dispersion scan (d-scan).^53,83^ The pulses have a maximum energy of ∼13 μJ after generation. After splitting and attenuation, the energy of a single pulse reaching the sample is ∼2.4 μJ, which could be varied in a controlled fashion. The pulse has an fwhm of ∼17.5 fs (2.6 optical cycles at 2 μm), and the electric field waveform depends on the carrier-envelope phase (CEP) in this regime. The spectral phase is compensated with a combination of ZnS wedges and chirped mirrors to compress the pulse, while the CEP is passively stabilized by the laser geometry.^53^ Since the SWIR pulses have incompletely compensated higher-order spectral phase, the temporal profile features the side lobes in the time domain (visible in Figure 1c). The polarization of the excitation pulses is also carefully controlled, taking into account the angular dependence of the nanowire field enhancement, and can be used to enhance the excitation of plasmon resonances by aligning the polarization in accordance with the orientation of the nanowire on the surface.

### Nanowires Structure and Sample Preparation

Silver nanowires (Ag NWs) were synthesized as in previous work.^84^ In the present work, we used nanowires with diameters around 32–35 nm and lengths of about 900–1100 nm. The wires were grown by seeded growth on premade pentatwinned gold nanobipyramids, which remain as central cores coated by silver, ensuring uniform growth along the axis. To evaluate the possible influence of the Au seed on the near-field distribution, additional FDTD simulations were performed with an included Au seed (coated with a 1 nm thin Ag layer^33,84^ and position variations along the axis). As a result, no significant change in the near-field enhancement was observed compared with pure Ag nanowires. For PEEM sample preparation, the solution containing a small amount of the cetyltrimethylammonium bromide (CTAB) surfactant to avoid aggregation and nanowires were diluted in deionized water, drop-cast onto clean substrates, rinsed, and dried in air. Substrates of n-doped Si and ITO were tested, but Si provided more stable and reproducible conditions for imaging with a lower background intensity. Samples were introduced into a vacuum shortly after the preparation to minimize additional surface oxidation and contamination. Representative SEM images of the samples and additional measurements performed with different excitation wavelengths are provided in the Supporting Information.

### Computational Model and Parameters

The electromagnetic response of the nanostructures was modeled by using finite-difference time-domain (FDTD) simulations carried out in the Ansys Lumerical FDTD Solutions package. A broadband total-field scattered-field (TFSF) source with a central wavelength of 1935 nm (155 THz) was used for imaging the field distribution, with a spectral width corresponding to a pulse duration of 17 fs. The excitation was incident along the *x*-axis at an angle of 65° with respect to the substrate normal. The polarization was aligned along the nanowire axis, which was consistent with the experimental geometry. The nanostructures were defined according to the experimental geometry as Ag nanowires with varied sharpness of tips placed on different substrates: indium tin oxide (ITO), glass (SiO_2_), and silicon with a thin 3 nm native oxide layer, the latter in accordance with the experimental conditions. The wavelength-dependent dielectric functions were taken from the tabulated values available in the software. Additional simulations including the Au seed (Ag-coated) show no significant change in the near-field response (see the Supporting Information). To analyze the observed local dynamical differences, we extracted the time-dependent electric fields at both nanowire ends from the FDTD simulations and computed corresponding IAC traces from the simulated time-domain fields (Figure 3a,b) in good agreement with the experimental data in Figure 2c.

## Supplementary Material



## Notes

Additional experimental details, SEM characterization of silver nanowires, substrate comparison, reproducibility analysis of interferometric autocorrelation traces and peak-shift extraction, measurements on additional nanowires, polarization-dependent photoemission, supplementary FDTD simulations for different nanowire geometries and substrates, influence of Au seed inclusion and incidence angle, time-dependent near-field maps, spectral response, hot-electron temperature dynamics simulation details, electromagnetic simulations of Ag nanowire plasmon dispersion, and details on estimates of fluence, peak intensity, and Keldysh parameter (PDF)
