# Supplementary material for: Ultrafast Near-Field Dynamics in Silver Nanowires Driven by Few-Cycle Short-Wave Infrared Pulses
Source: ACS Photonics. 2026 Mar 31;13(8):2248–59. doi: 10.1021/acsphotonics.6c00044 (PMC13088365; doi:10.1021/acsphotonics.6c00044)
Supplement: Supplementary file 1 [file ph6c00044_si_001.pdf]

Supporting information for: Time-resolved imaging of the near-field dynamics in silver nanowires excited by few-cycle short-wave infrared pulses

Nelia Zaiats [1*], Lukas Wittenbecher [1], Ivan Sytcevich [2], Anne-Lise Viotti[2], Chen Guo [2], Chandni Babu[3], Xiaolu Zhuo[4], Luis M. Liz-Marzáan[4,5], Eduardo J. C. Dias[5,6], F. Javier García de Abajo[7,8], Jan Vogelsang [9], Anne L'Huillier [2], Cord Arnold [2], and Anders Mikkelsen[1*]

[1]Department of Physics and NanoLund, Lund University, Sweden
[2]Department of Physics and Lund Laser Center, Lund University, Sweden
[3]Department of Chemistry and NanoLund, Lund University, Sweden
[4]CIC biomaGUNE, Basque Research and Technology Alliance (BRTA), Spain
[5]IKERBASQUE – Basque Foundation for Science, Spain
[6]POLIMA – Center for Polariton-driven Light-Matter Interactions, University of Southern Denmark, Denmark
[7]ICFO –The Institute of Photonic Sciences, The Barcelona Institute of Science and Technology, Spain
[8]ICREA – Catalan Institution for Research and Advanced Studies, Spain
[9]Institute of Physics, Carl von Ossietzky University of Oldenburg, Germany

*Email: nelia.zaiats@fysik.lu.se
anders.mikkelsen@fysik.lu.se

Contents

Characterization of silver nanowires . . . 2
Evaluation of hotspot dynamics . . . 3
Measurements on additional nanowires . . . 4
Polarization dependence of nanowire photoemission . . . 6
Nanowire model used in FDTD simulations . . . 7
Additional simulation results . . . 8
Details on time-dependent simulations . . . 11
Importance of different substrates . . . 14
Hot-electron temperature dynamics . . . 15
Electromagnetic simulations of Ag nanowire plasmon dispersion . . . 16
Fluence, peak intensity, and estimation of the Keldysh parameter . . . 17

Characterization of silver nanowires

The silver nanowires used in this study were first characterized by scanning electron microscopy (SEM) to confirm their typical dimensions.

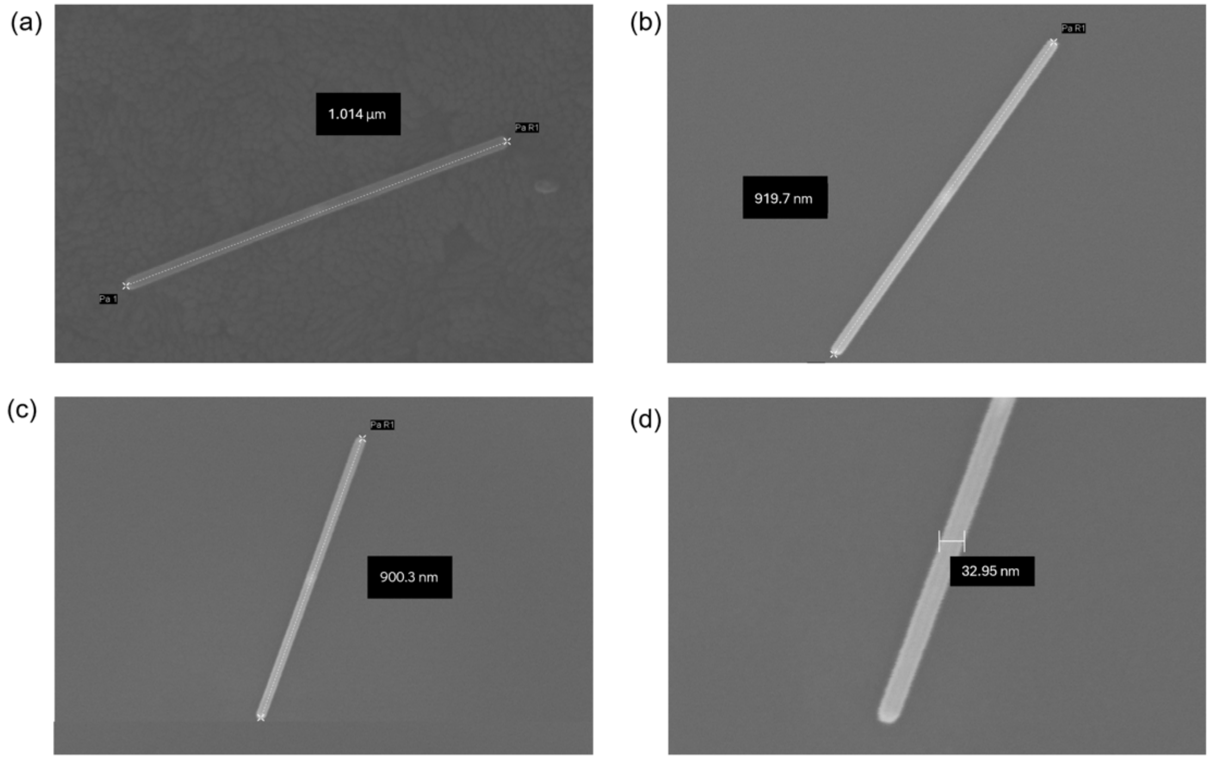

Figure S1: Scanning electron microscope (SEM) images of representative silver nanowires (NWs) used in this study. The nanowires exhibit lengths in the range of (a) 1015 nm, (b) 920 nm, and (c) 900 nm. (d) The diameter of the nanowires is ≈ 32 nm.

Figure S1 show SEM images of representative silver nanowires. The nanowires used in this study have lengths ranging from approximately 900 to 1050 nm and diameters of around 32 nm. Figure S1(a-c) show nanowires of different lengths, while (d) highlights the diameter across a nanowire. Nanowires are grown from gold by-pyramids at their core, which are subsequently coated with silver the growth process is described in [1]. There is a small amount of surfactants with estimated concentration below 0.5 mM. This surfactant prevents the aggregation of the nanowires. For sample deposition [2], following the steps mentioned in the Methods, most excess surfactant is removed, leaving only a thin layer of approximately 1-2 nm on the surface of each nanowire. This thin layer also helps to protect the nanowire from oxidation.

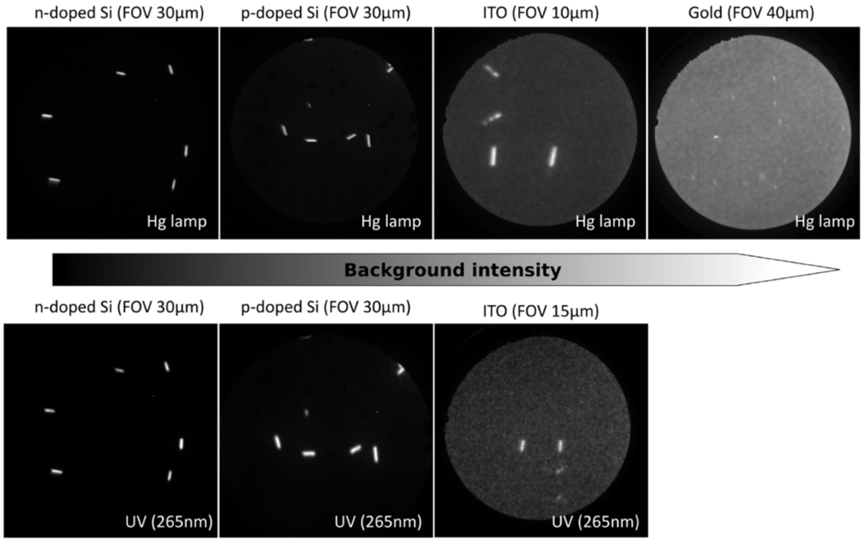

Figure S2: (a) Nanowires on different substrates: Si, Indium Tin Oxide (ITO) and Gold, imaged using a mercury (Hg) lamp (top row) and a UV light at 265 nm (bottom row).

Before time-resolved experiments were performed, careful substrate selection and testing were conducted. The choice of substrate is critical, since it not only modifies the local dielectric environment but also influences background photoemission [3]. The contrast images in Figure S2a help to distinguish between ITO-coated glass and Si substrates used for comparison. ITO and gold substrates exhibit significantly higher background emission. In contrast, n-doped and p-doped Si substrates show a very low and uniform background, allowing the nanostructures to stand out clearly with high contrast. Because Si yields minimal background intensity while maintaining good optical and electronic properties, it was selected as the substrate for the time-resolved measurements.

Evaluation of hotspot dynamics

To probe the ultrafast response, we measured interferometric autocorrelation (IAC) traces from the nanowires of Figure 2, three separate times to test reproducibility. The results were consistent across all datasets, showing fringes that confirm few-cycle excitations.

Small variations in the pulse shape are observed between the measurements, which can be explained by changes in the exact spectral distribution and thus temporal enveloped between pulses. However, these variations do not significantly affect the relative response of the individual hotspots along the nanowires. The relative intensity differences between the hotspots remain similar for all datasets, indicating that the local field enhancement is primarily governed by the nanowire geometry rather than by minor changes in the excitation conditions. Among the measured hotspots, spot 3 consistently exhibits the highest signal intensity. This result is reproducible across all datasets and is in good agreement with the spatial intensity distribution shown in Figure 2 of the main text, indicating the robustness of the observed hotspot response.

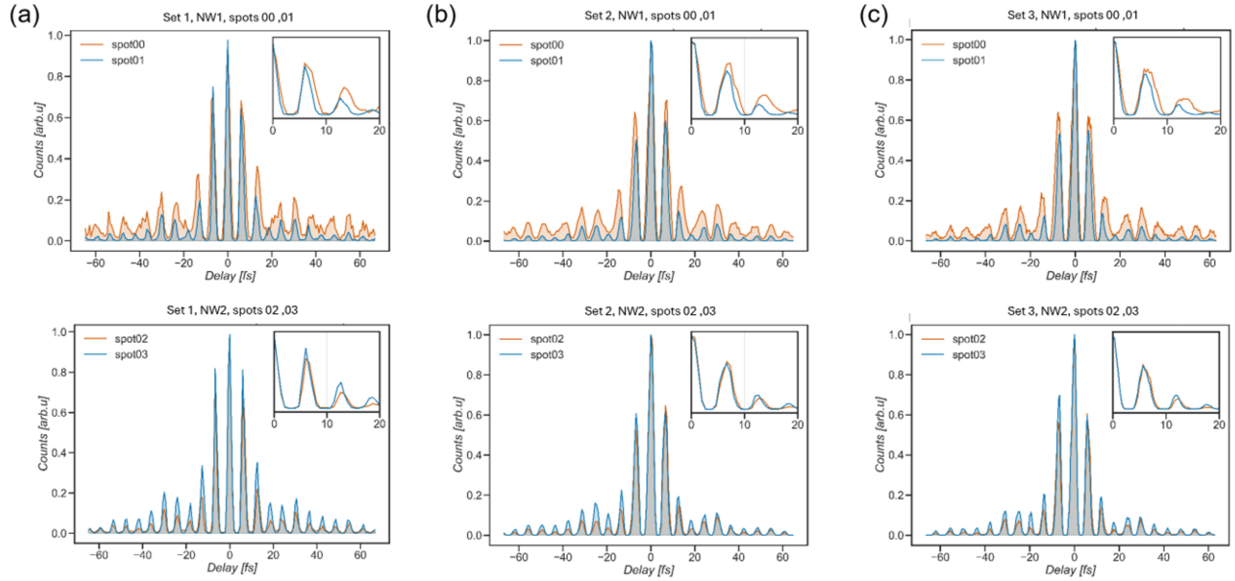

Figure S3: Normalized IAC traces for three independent datasets shown for hotspots 00-03 of the two nanowires discussed in the main text: (a) – dataset 1, (b) – dataset 2, (c) – dataset 3. Each series displays the same behavior when comparing the hotspots within a nanowire. Insets show a zoom of the early-time delays.

The other hotspots also show reproducible dynamic behavior, in Figure S3, for example spot 0 consistently exhibits a similar complex peak structures e.g. double features across all three datasets. This demonstrates that both the relative strength and the dynamics of the hotspots are repeatable even as the exact oscillatory shape of the light field changes slightly.

We also compared the shift of the pairs of hotspots for two different nanowires presented in Figure 2d of the main text for all three datasets and with the simulated nanowire response. To quantitatively extract these shifts, the IAC traces from each hotspot were first normalized and filtered (using a Savitzky-Golay filter) to reduce high-frequency noise and to provide robust peak localization when the delay sampling does not fall exactly at the peak center. The temporal maxima were then identified from the filtered traces, and the relative peak shift was obtained by subtracting the positions of the corresponding peak pairs (spot00-spot01 and spot02-spot03).

As we have performed three complete independent IAC measurements for the wires of Fig 2, we could also use this for an estimate of the uncertainty. The statistical uncertainty of the extracted peak positions could be determined as the standard deviation σ of each interference peak. The error bars are shown in Figure 2d. Based on this an averaged mean standard deviation across all peaks could be estimate to roughly 0.3 fs.

In addition, we tested an alternative processing approach based on Gaussian-convolution filtering applied to a densely interpolated version of the trace (figure below). In this method, firstly the measured IAC is interpolated to a finer delay grid, then each point is replaced by a weighted average of its neighboring points, with closer points contributing more strongly (Gaussian weighting). Peak positions are then determined from the maxima of this filtered trace. This approach also suppresses high-frequency noise while preserving the overall full peak shape. The results coincided with the results found using the method described above within the statistically obtained uncertainty.

Measurements on additional nanowires

Additional nanowires were measured to further validate the results. Some of these nanowires were oriented at different angles with respect to the incident light, while others were located very close to neighboring wires, which could lead to artificially enhanced local fields due to coupling effects. For the analysis, we

therefore selected nanowires that were sufficiently isolated, ensuring that the observed hotspots could be clearly identified and attributed to individual nanowires.

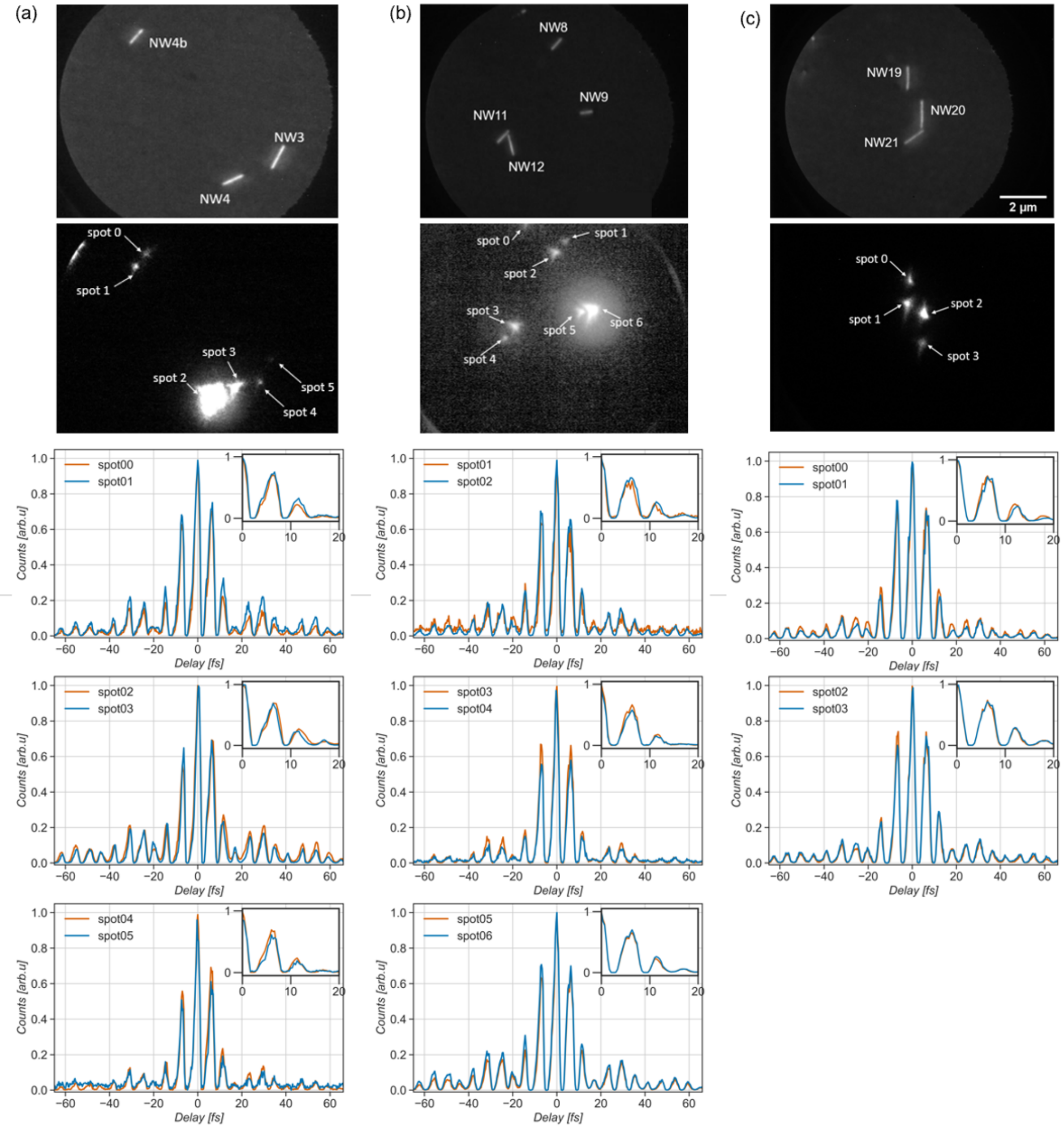

Figure S4: Additional silver nanowires measured. (a-c) The top panels show PEEM images of the selected nanowires recorded with Hg lamp and SWIR laser pulses, with the corresponding hotspots. Bottom panels display the normalized IAC traces for each hotspot pair with zoomed-in insets for shorter delay times. Nanowires 19–21 (panel c) were measured under s-polarized excitation.

The additional nanowires shown in Figure S4 exhibit the same general trends as observed in the main text. Each nanowire shows pronounced hotspots at both ends. While the overall photoemission intensity varies between hotspots, some being brighter than others, the temporal shape of the IAC traces remains similar. Several hotspots also show a double-peak structure in the IAC, whereas others exhibit a more symmetric single peak. The general observed temporal response is robust and does not depend strongly on

the individual nanowire or minor changes in the measurement conditions. As in the main text, for some traces the dynamic response in the two ends of the wire is almost identical, while others exhibit higher peak shifts in the IAC between the two wire ends. For example, in Figure S4a, the IAC traces of nanowire 3 differ from those of nanowire 4b, indicating that with the same dynamic profile of the beam, differences across the same image are related to differences between the individual wires. In a few cases, we also changed the polarization of the excitation light. For nanowires 19-21 (Figure S4c), which are oriented vertically and therefore parallel to the p-polarized excitation, the emission becomes significantly brighter. This behaviour is expected, as aligning the long axis of the nanowire with the incident polarization drives the longitudinal plasmon modes [4, 5], resulting in stronger hotspot emission. In contrast, when the nanowire is oriented perpendicular to the polarization direction (e.g., NW12 for s-polarization or NW21 for p-polarization), the emission is much weaker. Because of this clear polarization dependence, for the data shown in the main text we selected nanowires that are aligned with the s-polarized excitation, ensuring a consistent and comparable excitation geometry.

Polarization dependence of nanowire photoemission

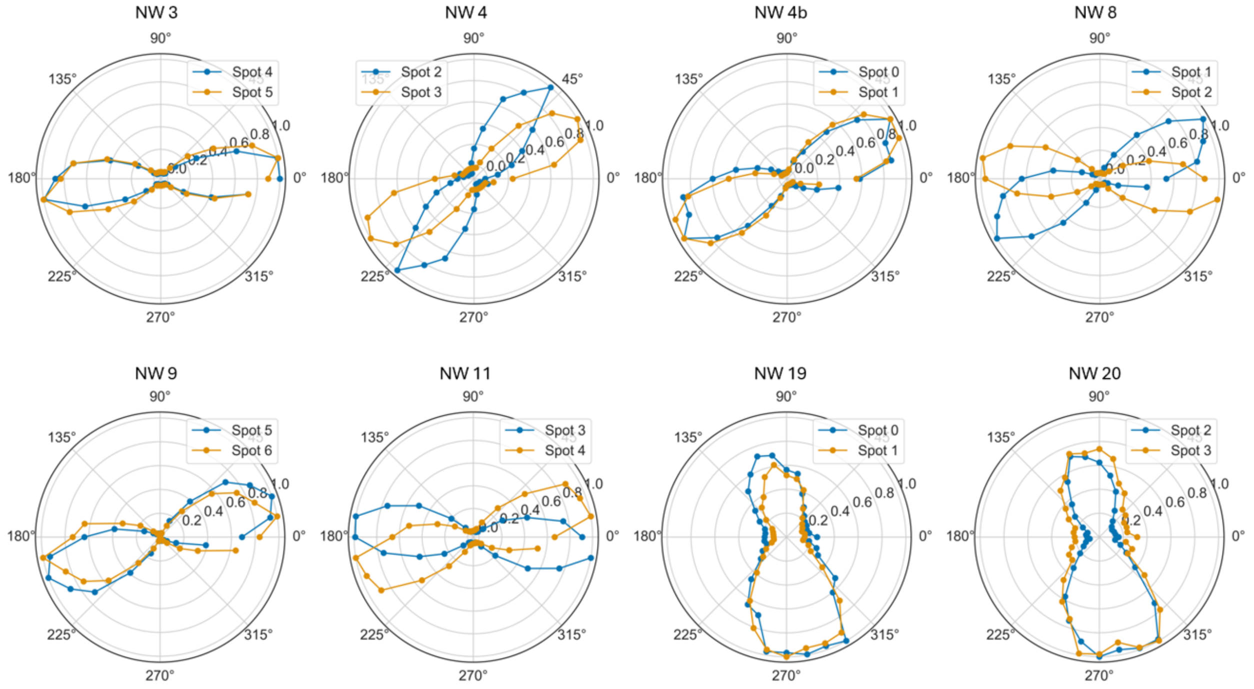

Figure S5: Polarization dependence of the normalized emission for selected nanowires from Figure S4. Each plot shows the photoemission intensity of the indicated hotspots as a function of incident polarization angle.

Polarization-dependent measurements of the emission from the silver nanowires were measured (Figure S5). As can be seen the emission depends on the polarization of the incident field in a similar fashion, as would be expected [6]. For all nanowires we observe that the maximum mostly occurs when the electric field is aligned along the long axis of the nanowire. This behaviour is consistent across different nanowires and across hotspots, showing that the excitation efficiency is affected by the geometric alignment between the nanowire and the driving field. Changing polarization of the incident light, both nanowires 19 and 20 are oriented nearly vertically in the PEEM images, and their corresponding polar plots show clear maxima around the vertical polarization direction ($\sim 90^\circ/270^\circ$), also consistent with excitation of the longitudinal plasmon mode with p-polarized light. In all cases, the emission drops when the polarization is rotated towards the axis, where the electric field becomes perpendicular to the wire.

Nanowire model used in FDTD simulations

To model the near-field response around the nanostructure, we constructed in Lumerical [7] a 3D geometry consisting of a silver nanowire. The nanowire length and the tip geometry were varied to study how the near-field response changes with different termination shapes. In addition to Si/SiO_2, we also tested an ITO layer as an additional substrate configuration for comparison. The mesh is refined around the entire nanowire, with 2 nm grid size to ensure accurate resolution of the localized fields. Time-domain and frequency-domain monitors of different dimensions (1D, 2D, and 3D) are positioned along the wire, at the tip, and the substrate interface. These monitors allow us to track the field evolution, spectral content, and local intensity distribution in different regions of interest.

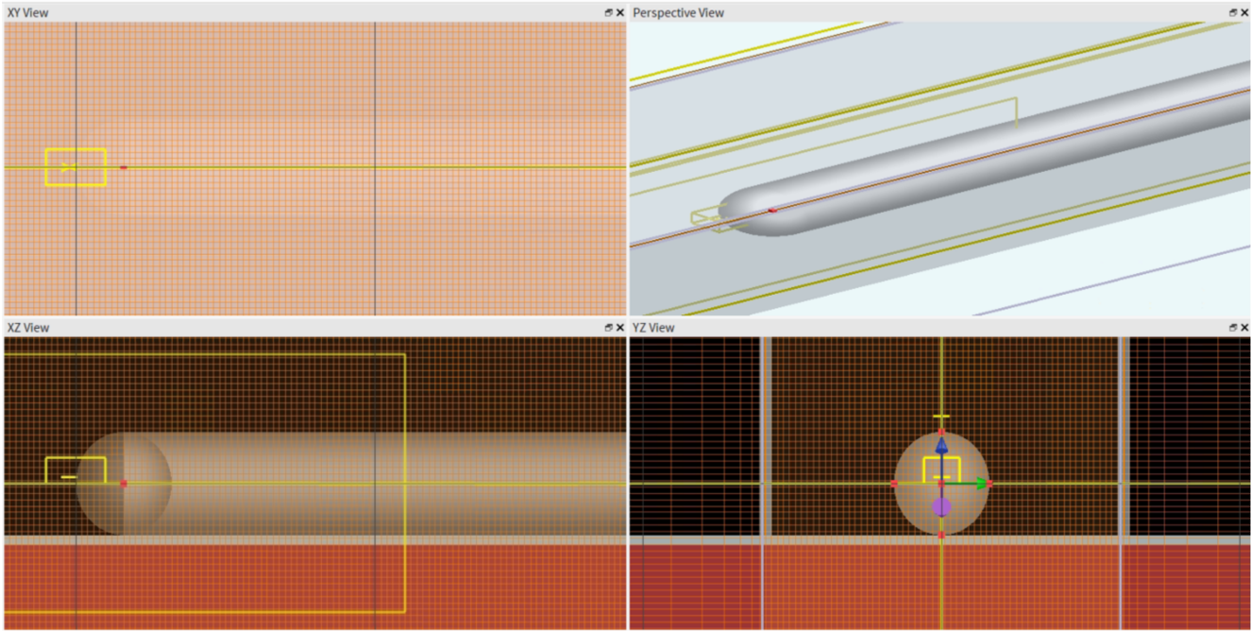

Figure S6: The simulation geometry used in the FDTD calculations. The XY, XZ, and YZ projections show the alignment of the mesh and monitoring regions with the nanowire axis.

The modelling conditions are similar to the PEEM experiments, where a pulsed excitation is used to generate a time-dependent field (Figure S6). This time-dependent field is then Fourier transformed to access the corresponding quantities in the frequency domain. A planar wave excitation is applied to extract the IAC traces, while a total-field scattered-field (TFSF) source is included to correctly capture the response of the finite nanowire geometry. Both sources are aligned along the axis of the structure with a 65-degree angle towards the normal to reproduce the experimental setup.

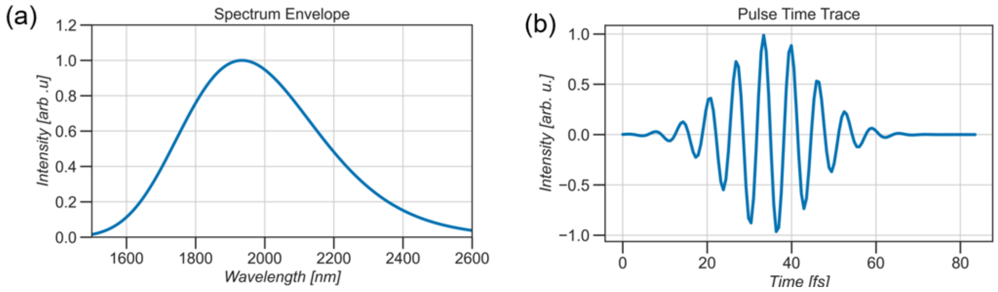

Figure S7: Spectrum (a) and time-domain trace (b) of the illumination pulse used in the FDTD simulations.

For the simulations, we use a spectrum centered around 1950 nm with a bandwidth spanning 1600–2500

nm. This corresponds to a Gaussian pulse with central frequency and duration determined by the spectrum and pulse length, which is the closest approximation to the pulses used in the experiment. The corresponding time-domain pulse is obtained through a Fourier transform (Si/SiO_2). This "ideal" pulse, gives a clean temporal profile without additional features. While in the experiment, the broad bandwidth of the SWIR pulse causes a spectrum that shows variations in intensity across the bandwidth (Figure 1b). The time-domain pulse in the measurements setup (Figure 1c) also contains side lobes, which are discussed in detail in the main text. To model the near-field response of the nanowire closer to the experimental data, we implemented wavelength-dependent dielectric functions: Ag, Si, Si/SiO_2, and ITO [7, 8]. Silver has a dispersive model that captures its strongly negative real permittivity and increasing losses toward longer wavelengths. The Si substrate was included with its high refractive index and very low absorption in the SWIR. A thin 3 nm Si/SiO_2 layer was added with its nearly constant permittivity and negligible losses. We also tested ITO as an alternative surface layer. In this spectral range, neither pure Si/SiO_2 nor ITO couples strongly to the nanowire's plasmonic mode. As a result, overall, the main material-driven effects in the simulations come from silver and the presence of the Si substrate.

Additional simulation results

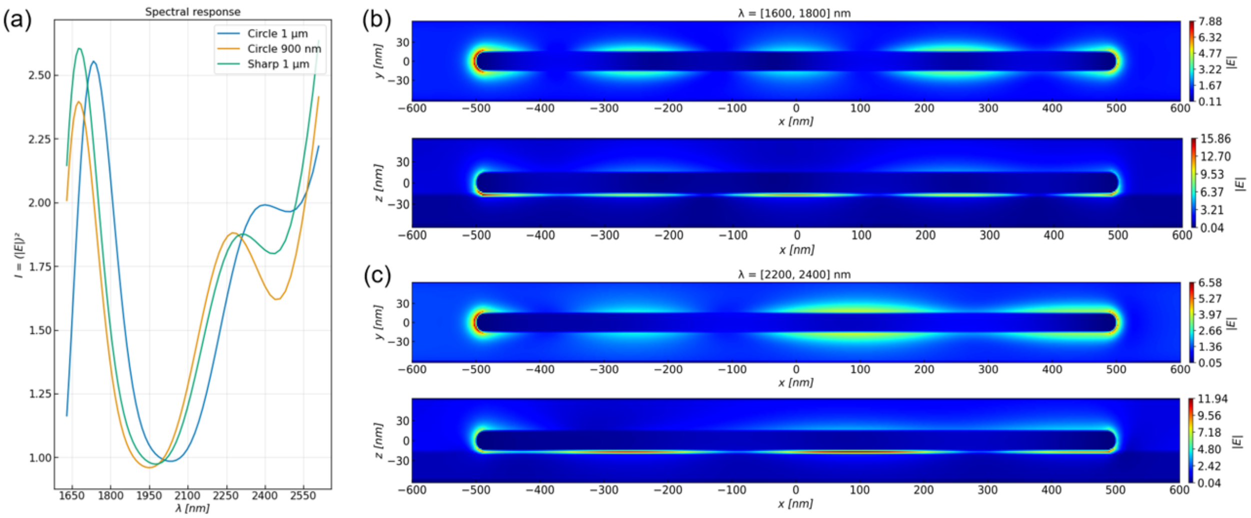

Figure S8: Spectral near-field response over the excitation range from 1700 to 2600 nm light wavelength. (a) Spectral response integrated along the full nanowire length for 3 different shapes. (b) Near-field distributions at wavelengths between 1600 and 1800 nm, and (c) near-field distributions at wavelengths between 2200 and 2400 nm in top and side views.

To characterize the optical behavior of the nanowires, we evaluated the spectral response over the full simulation wavelength range (Figure S8a). At lower wavelengths (∼1600–1800 nm), the wire supports a weak higher-order longitudinal mode, which produces only weak field enhancement. At wavelengths corresponding to the first spectral peak, five distinct hotspots can be observed along the nanowire (Figure S8b), indicating the excitation of a higher-order plasmon mode. As the energy increases, the response passes through a pronounced minimum around ∼2000 nm, where destructive interference along the wire reduces the field.

At even higher wavelengths, the field increases again. In this regime, the field distribution changes and only four hotspots are observed along the wire, as shown for wavelengths around 2200–2400 nm (Figure S8c). Despite the presence of multiple hotspots, the strongest field enhancement remains localized at the wire ends.

A comparison of the three geometries shows that the overall spectral shape is similar for all wires, but the resonance positions and enhancement strengths depend on the nanowire length and the shape of the tips. The shorter 900 nm nanowire exhibits a small spectral shift of both the minimum and the main resonance toward shorter wavelengths compared to the 1 μm nanowires, consistent with its reduced length. In contrast, the 1 μm circular nanowire is shifted toward longer wavelengths compared to the other geometries. The 1 μm

sharp nanowire lies in between, with resonance positions close to those of the circular wire. These shifts indicate that both the nanowire length and the tip geometry influence the spectral response.

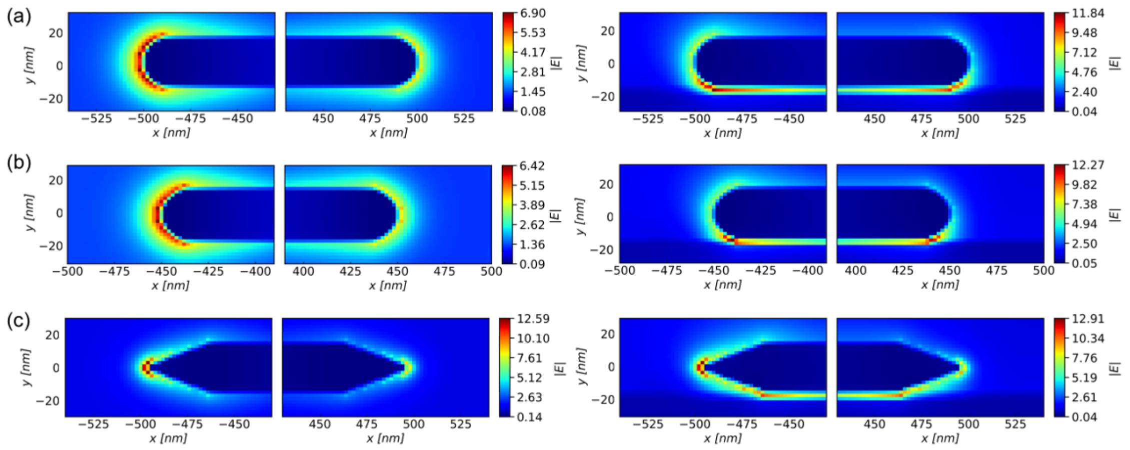

Figure S9: Top and side views of the simulated near-field distribution for three different nanowire geometries summed for excitation wavelengths between 1600 and 2400 nm: (a) a 1 μm circular nanowire, (b) a 900 nm circular nanowire, and (c) a 1 μm nanowire with sharp tips.

To investigate the influence of nanowire geometry, we compare the near-field response of three different nanowire structures in both top and side views (Figure S9). The corresponding interferometric autocorrelation (IAC) traces for these geometries are shown in Figure 3b of the main text. Despite identical excitation conditions, the spatial localization of the field strongly depends on the tip shape. The circular nanowires exhibit relatively broad and symmetric hotspots at the left end of the wire, with only minor variations in strength depending on the field. In contrast, the sharp-tip nanowire produces a much more localized and intense hotspot, highlighting the strong effect of tip geometry.

The side-view reveals that a significant fraction of the field is located underneath the nanowire, close to the substrate, indicating strong coupling between the nanowire and the substrate. For all geometries, the field strength is higher at the left end of the nanowire. This asymmetry likely arises from the incident light propagating from the right side, as will be discussed further.

These results demonstrate that both the nanowire length and the tip shape play an important role in determining the spatial distribution and strength of the near-field hotspots.

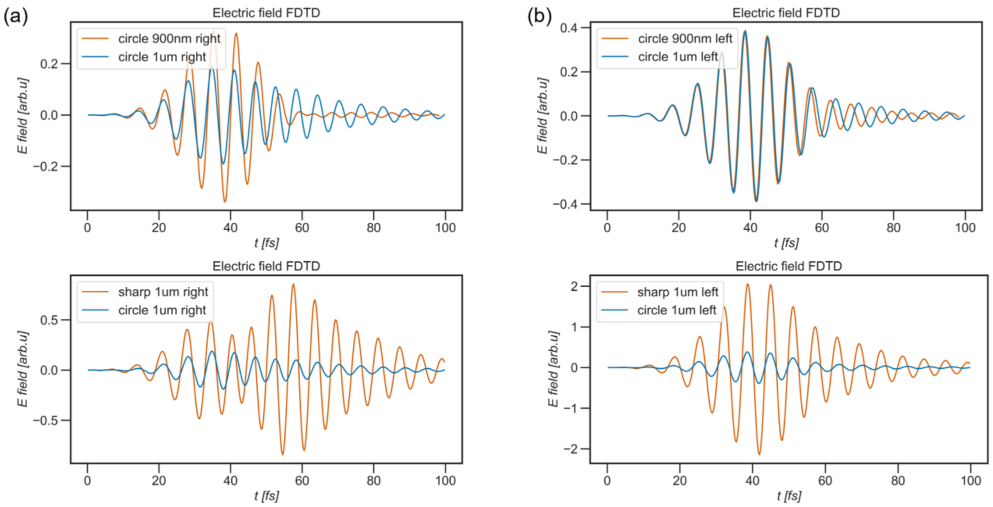

Figure S10: Time-domain electric fields extracted from the simulations at the left and right ends for a 1 μm circular nanowire, a 900 nm circular nanowire, and a 1 μm nanowire with sharp tips. Panel (a) compares the electric fields at the right ends of the nanowires, while panel (b) shows the fields at the left ends.

We compare the left and right ends of the time-domain fields for the different geometries (Figure S10). The differences turn out to be minimal, and are mostly visible in the electric field, for the left nanowire ends. The plasmonic fields at the left ends, shown in Figure S9, are almost identical in shape for all three geometries. The main difference is the field strength, while the temporal dynamics and oscillation patterns remain very similar.

This behavior can be explained by the illumination geometry. The SWIR excitation arrives from the right side at an oblique angle and therefore directly interacts with the right nanowire tip. The left end, however, is located on the opposite side and is not directly illuminated. Instead, the excitation launches a surface plasmon wave that propagates along the nanowire toward the left end. As a result, the local field dynamics at the left end are dominated by this propagating mode rather than by the local tip geometry.

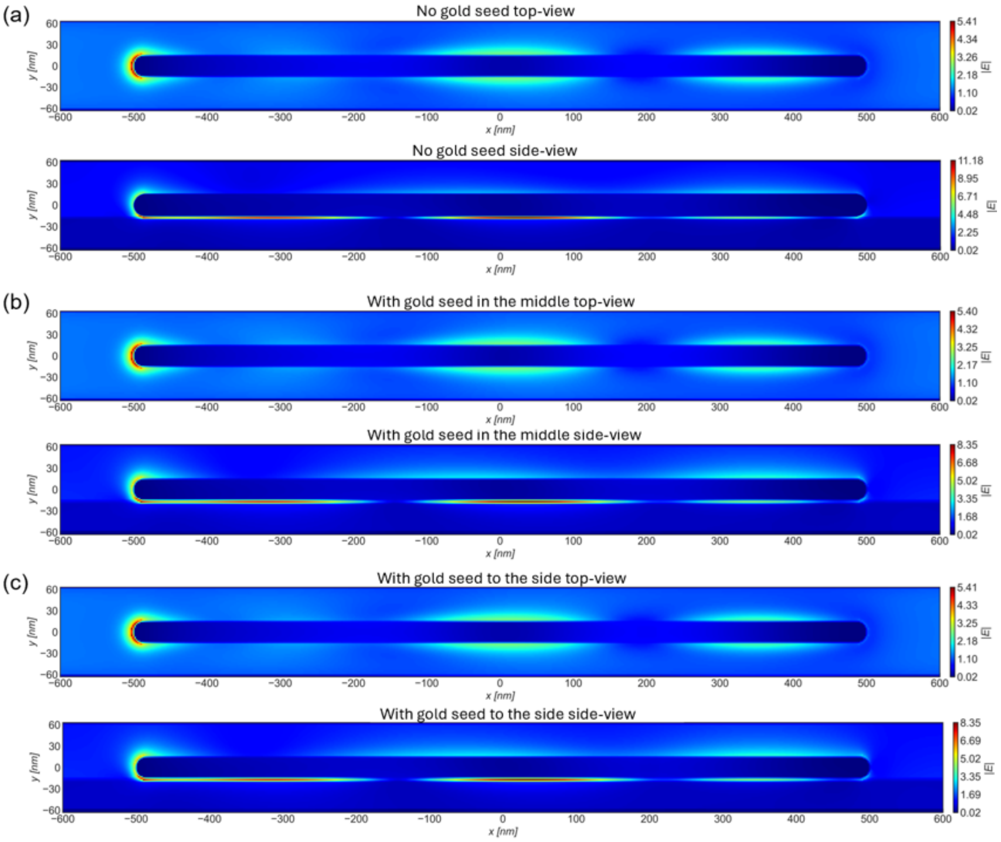

Figure S11: Simulated influence of a Au seed inside the Ag nanowire on the near-field response at 2100 nm incidence. Near-field maps (top view) at the central wavelength ($\lambda = 2100$ nm) for (a) an Ag nanowire without Au seed, (b) the same nanowire including Au seed coated by a 1 nm Ag at the center, and (c) the same configuration with the seed displaced 200 nm along the nanowire axis.

We additionally performed numerical simulations including a Au core inside the Ag nanowire. This Au seed was modeled as a 30 nm diameter spherical inclusion coated by a 1 nm thick Ag layer, reflecting the experimental growth conditions. Three configurations were considered: Figure S11a - a nanowire without Au seed, Figure S11b - a nanowire with the Au seed positioned in the center, and Figure S11c a nanowire with the Au seed displaced by 200 nm from the center. In all cases, the calculated near-field enhancement at the Ag top surface remains essentially unchanged. Even though there is a somewhat stronger substrate coupling in the presence of the gold seed, the top-view figures look identical. Neither the spatial field distribution nor the temporal plasmonic dynamics show qualitative differences upon inclusion or displacement of the Au particle. This behavior can be understood as due to the very similar dielectric functions of Au and Ag in the relevant spectral range. Furthermore, since the Au seed is fully covered by Ag, the surface composition and thus the work function relevant for photoemission remain unchanged.

Details on time-dependent simulations

To visualize the time-dependent response of the nanowire, we simulated the near-field distribution as a function of time delay, rather than only as a function of wavelength.

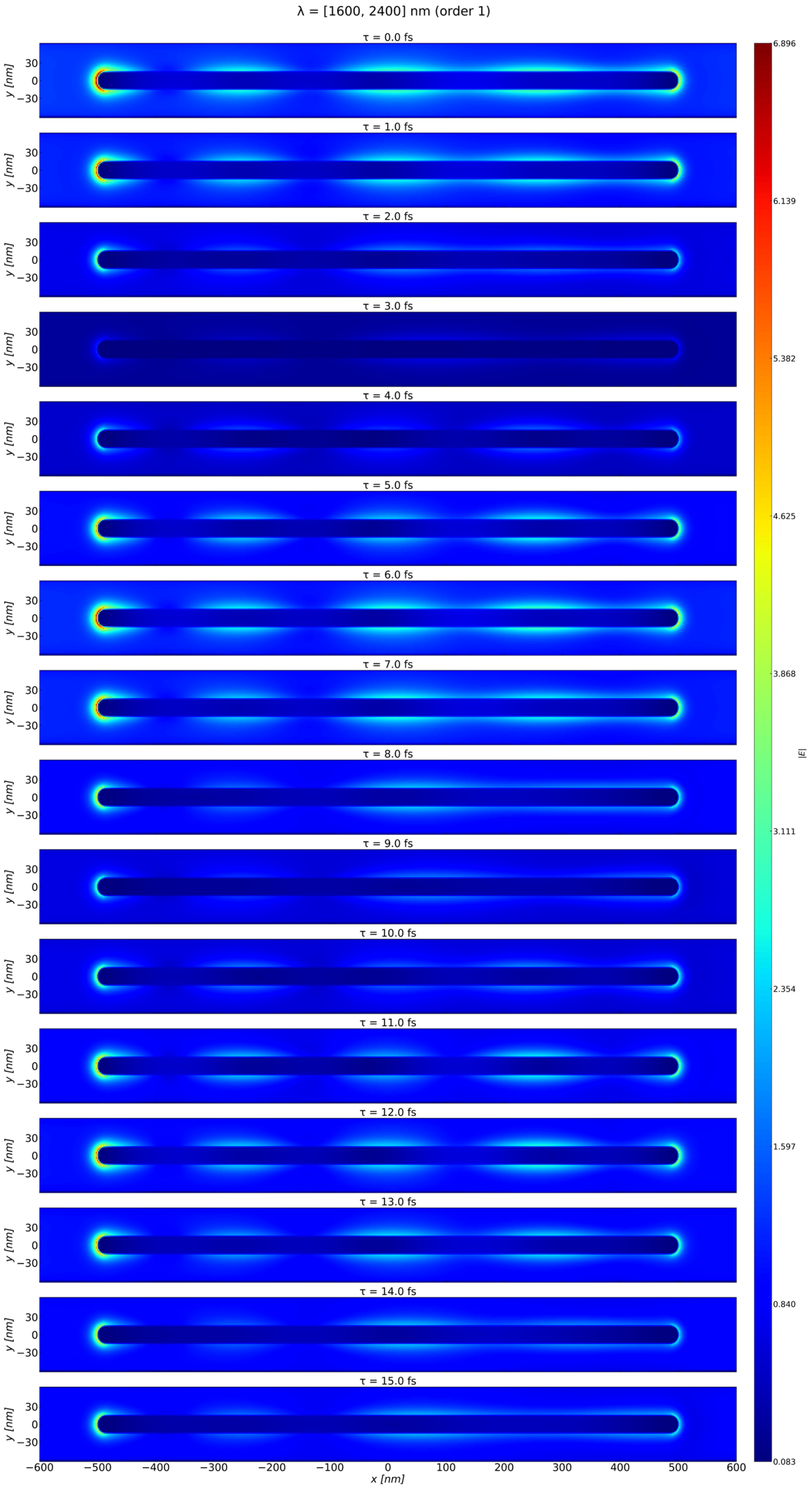

Figure S12: Simulated time-dependent near-field distribution of a 1 μm circular nanowire simulated in Lumerical. The field maps are shown for delays from 0 to 15 fs in steps of 1 fs, for excitation wavelengths between 1600-2400 nm.

Figure S12 shows how the plasmonic modes along the nanowire evolve over time. The spatial field distribution changes strongly with delay, indicating an oscillation of the excited mode. At early delays (around 0–2 fs), a strong field is observed at the nanowire ends and along the wire. Around a delay of approximately 3 fs, the near-field intensity is reduced and almost disappears. At later delays, the field reappears and the field structure becomes visible again. This behavior is consistent with the IAC traces, where the signal oscillation plotted, also suppressed (3, 9, 15 fs) and reappear (7, 12 fs) around similar delay values. At some delays, small shifts in the spatial position of the plasmonic modes along the nanowire can be observed. These shifts are most pronounced at delays where the signal is suppressed (3, 9 fs). This behavior can be explained by the fact that different spectral components of the broadband excitation pulse contribute with different relative phases at different delays. As a result, different fractions of the pulse spectrum dominate the excitation at these times, leading to slight changes in the excited plasmon mode pattern.

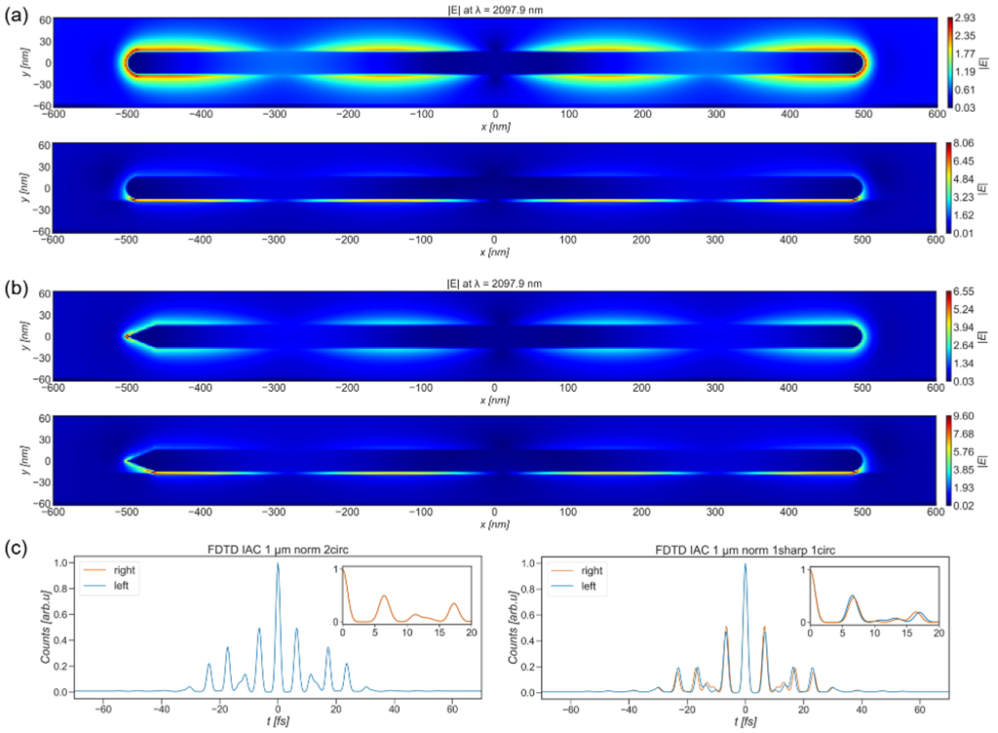

Figure S13: Simulated near-field distribution under normal incidence excitation at the central wavelength 2100 nm for (a) 1 μm nanowire with symmetric circular ends and (b) nanowire of 1 μm with one end sharper.

We also considered the influence on the angle of incidence of the SWIR pulses on the dynamics. In the experiments, the SWIR pulses impinge the sample at 65° with respect to the surface normal. For a wire of length $L \sim 1$ μm aligned with the in-plane projection of the beam, the maximum arrival-time difference is $\Delta t_{\text{geo}} \approx (L/c) \sin 65^\circ \approx 3$ fs. Because pump and probe pulses propagate collinearly in our experiment, this retardation contributes only a constant offset to the local time zero between the two ends and does not generate delay-dependent phase evolution in the interferometric autocorrelation (IAC) peak shifts. However, to assess how the illumination angle influences the near-field distribution and symmetry, we performed additional FDTD simulations using normal incidence excitation while keeping all other parameters identical. Figure S13 shows representative near-field maps under normal incidence. Compared to oblique incidence (main text, Figure 3), normal incidence yields a symmetric field distribution with respect to the nanowire center as well as symmetric IAC traces. This is a very reasonable behaviour as the wire-light source system is completely symmetric in the long axis direction of the wire. Thus, no differences in the dynamic field response of the two ends should be expected. However, when a geometric asymmetry is introduced, for example, when one end is sharper than the other (as in Figure S13b), the field enhancement becomes stronger

at the sharper end even under normal incidence. From the corresponding IAC traces in Figure S13c, it is further evident that the temporal response is no longer perfectly symmetric between the two ends. Although the excitation is normal to the substrate, the sharper tip exhibits a slightly modified oscillation amplitude and a phase deviation compared to the rounded end. This indicates that geometric asymmetry alone can induce differences in both the amplitude and temporal evolution of the local plasmonic response. The dominant near-field enhancement remains localized at the nanowire ends also at normal incidence. Thus, while oblique incidence can introduce an overall asymmetry between left and right ends through the illumination direction, it does not change the qualitative conclusion that the strongest emission originates from end-localized field maxima.

Importance of different substrates

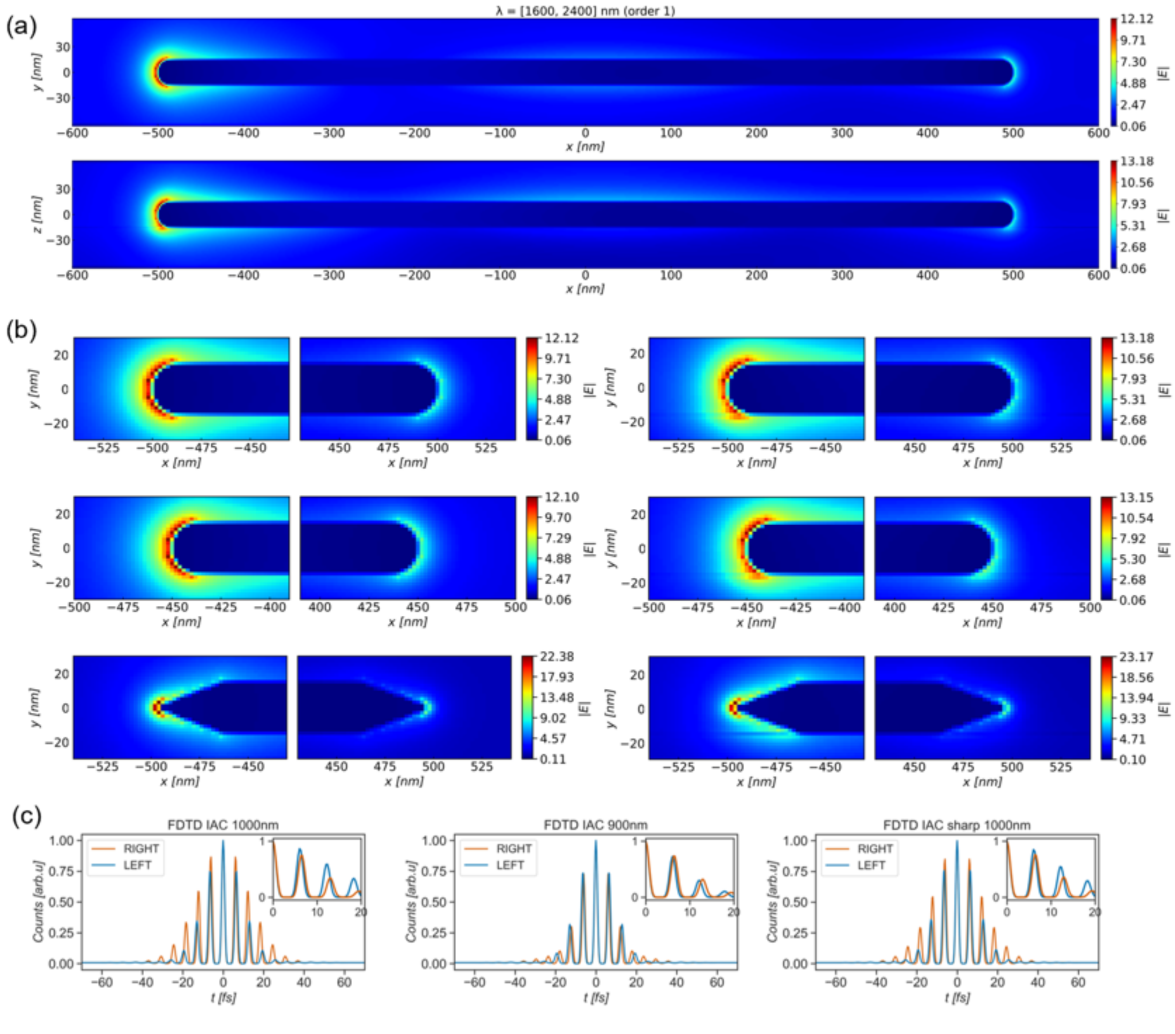

Figure S14: Simulated near-field distribution for the nanowires on ITO substrate at 1600-2400 nm range. (a) Top and side views for the full 1 µm nanowire. (b) Zoomed at the ends of the 1 µm circular, 900 nm circular, and 1 µm sharp nanowires, (c) together with the corresponding IAC traces.

In contrast to the Si/SiO_2 case shown in the main text, the nanowire placed on ITO (Figure S14) exhibits almost no substrate coupling, unlike the case of Si shown in Figure S9. The field pattern resembles that of a free-standing nanowire, with the strongest enhancement confined to the tips and only weak interaction with the substrate. In addition, fewer plasmonic modes are enhanced. This behavior is consistent with the

dielectric properties of ITO in the SWIR range, which are much closer to those of air than to Si and therefore do not induce additional plasmon modes through substrate–wire interaction.

From the close-up field distributions in Figure S14b, differences are observed between the sharp and circular nanowires at the wire ends. The sharp tips show a more localized and stronger near-field compared to the circular tips, indicating a strong effect of tip geometry on the spatial field distribution. In contrast, the IAC traces in Figure S14c show that the temporal dynamics of the two 1 μm nanowires are nearly identical, despite their different tip shapes. The 900 nm nanowire, however, exhibits a clearly different IAC response. In addition, the IAC traces from the two ends of the 900 nm wire show only a small relative shift in the oscillations.

Hot-electron temperature dynamics

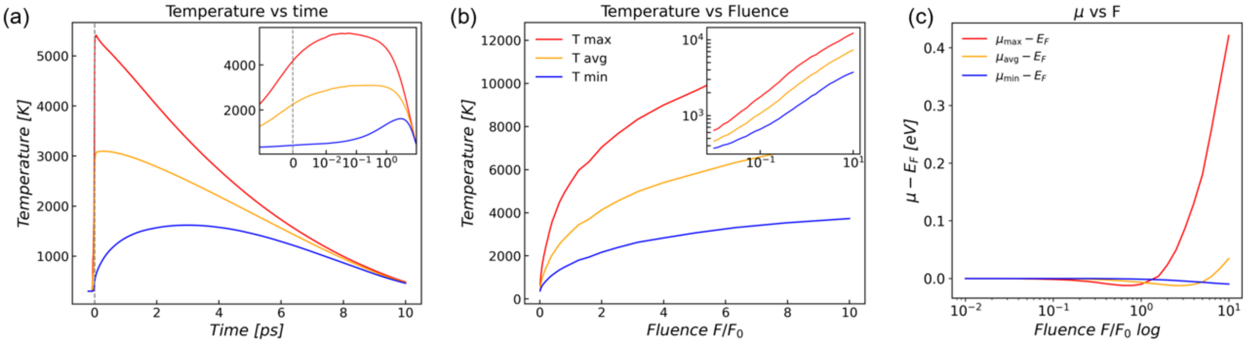

Figure S15: Simulated electron temperature dynamics and fluence dependence in silver nanowires. (a) Time evolution of electron temperatures across the nanowire at a fluence of 18 mJ/cm^2. Inset show the log scale and time 0 is indicated by grey dashed line. (b) Electron temperatures as a function of incident laser fluence with a log scale inset. (c) Corresponding shift of the chemical potential relative to the Fermi level as a function of fluence.

To estimate the effect of laser heating on the silver nanowires, we simulate the spatiotemporal dynamics of the electron temperature $T_e(\mathbf{r}, t)$ across the nanowire position $\mathbf{r}$ and time t using the two-temperature–type diffusion equation [9, 10]

$$c_e \frac{\partial T_e(\mathbf{r}, t)}{\partial t} = p_{\mathrm{abs}}(\mathbf{r}, t) + \nabla \cdot [\kappa_e \nabla T_e(\mathbf{r}, t)] + g_{el} [T_e(\mathbf{r}, t) - T_0], \quad (1)$$

where c_e and κ_e are the electronic heat capacity and thermal conductivity of silver, respectively [11]. The lattice temperature is fixed at $T_0 = 300$ K, which we approximate as equal to the ambient temperature due to the much larger lattice heat capacity compared to the electronic contribution [10]. The parameter g_{el} denotes the electron–phonon coupling constant [11], and p_{abs} is the absorbed power density.

The absorbed power density is assumed to be separable into spatial and temporal components,

$$p_{\mathrm{abs}}(\mathbf{r}, t) = p_{\mathrm{abs}}(\mathbf{r})\, T(t), \quad (2)$$

where the temporal profile $T(t)$ corresponds to the pulse envelope shown in Fig. 1c. The spatial dependence is obtained from frequency-domain near-field simulations of the field enhancement $\varepsilon(\omega, \mathbf{r}) = E(\omega, \mathbf{r})/E_0$ for an incident field of amplitude E_0 at angular frequency ω, and is given by [12]

$$p_{\mathrm{abs}}(\mathbf{r}) = \frac{\omega F_0}{c\, \Delta t} |\varepsilon(\omega, \mathbf{r})|^2 \, \mathrm{Im}\{\varepsilon_{\mathrm{Ag}}(\omega)\}, \quad (3)$$

where $F_0 = (c\varepsilon_0/2)\, |E_0|^2\, \Delta t$ is the incident pulse fluence, $\Delta t = \int T(t)\, dt \approx 21$ fs is the effective pulse duration, and $\varepsilon_{\mathrm{Ag}}(\omega)$ is the dielectric function of silver.

For simplicity, and for the purpose of estimating the temperature dynamics, the absorbed power is evaluated at the central pulse frequency $\omega = 2\pi c/\lambda$, corresponding to a wavelength $\lambda = 2.1$ μm.

Due to strong near-field enhancement at the nanowire surface and tips, the maximum electron temperature rises rapidly within a few femtoseconds and exceeds 5000 K (Figure S15a). The system subsequently cools on a picosecond timescale via electronic heat diffusion and electron–phonon coupling, becoming nearly spatially uniform after approximately 8 ps and returning toward ambient temperature after ~ 10 ps.

As shown in Figure S15b, the electron temperature increases strongly with fluence and can reach values as high as 12000 K at the nanowire ends. However, for the fluence used in the experiments, the resulting chemical potential shift relative to the Fermi level is very small, $\mu - E_F \approx -0.01$ eV (Figure S15c).

These results demonstrate that although substantial electron heating occurs—particularly at the nanowire tips—the associated chemical potential shifts and thermal broadening are too small to account for the experimentally observed reduction in the effective photon number. This indicates that photothermal effects are not the dominant mechanism underlying the nonlinear photoemission behavior observed in the experiments.

Electromagnetic simulations of Ag nanowire plasmon dispersion

To support the interpretation of the experimental data in terms of plasmonic modes of Ag nanowires, we performed electromagnetic simulations of the dispersion relation of infinitely long Ag nanowires using a boundary-element method (BEM) [13, 14].

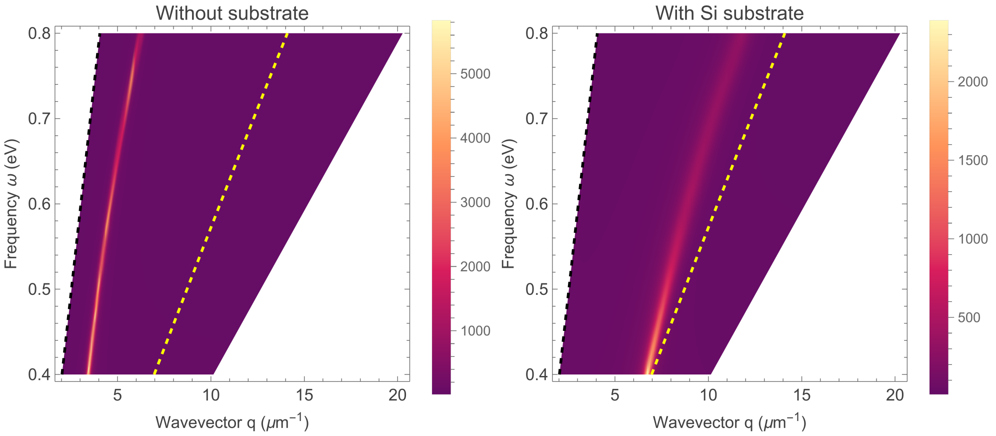

Figure S16: Calculated local density of optical states (LDOS) per unit length for an infinite Ag nanowire, resolved as a function of photon energy $\hbar\omega$ and longitudinal wave vector q. Left: nanowire in air. Right: nanowire on a Si substrate. Bright ridges correspond to guided plasmon modes. Black and yellow dashes lines are the dispersion of light in air and Si, respectively.

The LDOS per unit length for an electric dipole positioned just above the nanowire surface was calculated as a function of photon energy $\hbar\omega$ and longitudinal wave vector q, enabling identification of the guided plasmon modes supported by the nanowire across the UV–Vis–NIR spectral range. Two geometries were considered: an Ag nanowire in air and an Ag nanowire on a Si substrate.

In the dispersion maps shown in Figure S16, the longitudinal wave vector q is directly related to the plasmon wavelength, near-field enhancement, and degree of confinement. The LDOS is represented as a color scale, where bright regions indicate strong coupling to guided plasmon modes. The single bright dispersive branch corresponds to the fundamental guided plasmon mode of the nanowire. The variation in intensity along this branch reflects changes in mode confinement, group velocity, and damping. The presence of the Si substrate increases mode confinement and reduces the group velocity, leading to an enhanced LDOS at lower energies while simultaneously broadening the mode due to increased dielectric loss associated with the high refractive index of Si.

At the SWIR photon energy used in the experiments (centered at 0.62 eV), the dispersion yields $q \approx 4.8\ \mu\text{m}^{-1}$ in air and $q \approx 9.1\ \mu\text{m}^{-1}$ on Si, corresponding to plasmon wavelengths $\lambda_p = 2\pi/q$ of approximately 1.3 μm (air) and 0.7 μm (Si). This demonstrates the substantially stronger confinement of the guided mode in the substrate-supported nanowire.

Fluence, peak intensity, and estimation of the Keldysh parameter

To estimate the strong-field parameters in our experiment, we start from the average output power of the SWIR source, $P_{\text{laser}} = 1.9$ W, operated at a repetition rate of $f = 200$ kHz. This corresponds to a pulse energy at the laser output of

$$E_{\text{laser}} = \frac{P_{\text{laser}}}{f} \approx 9.5\ \mu\text{J}. \tag{4}$$

Because the pulses were sent through an interferometer and further attenuated to suppress space-charge effects in the photoemission electron microscope, the exact pulse energy at the sample is not known. Based on a measured average power of $P_{\text{avg}} \approx 475$ mW under the experimental conditions, we estimate the pulse energy at the sample to be

$$E_p = \frac{P_{\text{avg}}}{f} \approx 2.4 \times 10^{-6}\ \text{J}. \tag{5}$$

The focal spot at the sample is characterized by an elliptical beam profile with full widths $a = 201\ \mu$m and $b = 85\ \mu$m. Approximating the intensity distribution as an ellipse, the effective illuminated area is

$$A = \frac{\pi}{4} ab \approx 1.34 \times 10^{-8}\ \text{m}^2. \tag{6}$$

The corresponding peak fluence is then

$$F = \frac{E_p}{A} \approx 1.8 \times 10^{-2}\ \text{J}\,\text{cm}^{-2}. \tag{7}$$

Using a pulse duration of $\tau = 17.5$ fs (corresponding to approximately 2.5 optical cycles at the central wavelength) and treating the temporal envelope as a short pulse, we estimate the peak intensity as

$$I_{\text{peak}} \approx \frac{F}{\tau} \approx 0.67 \times 10^{12}\ \text{W}\,\text{cm}^{-2}. \tag{8}$$

The corresponding peak electric field amplitude in free space follows from

$$I_{\text{peak}} = \frac{1}{2} c\varepsilon_0 E_0^2, \tag{9}$$

where c is the speed of light and ε_0 is the vacuum permittivity. This yields

$$E_0 = \sqrt{\frac{2I_{\text{peak}}}{c\varepsilon_0}} \approx 2.2 \times 10^9\ \text{V}\,\text{m}^{-1}. \tag{10}$$

Assuming no additional near-field enhancement at the sample surface ($E_{\text{loc}} = E_0$), this value is used as the local driving field.

For a central wavelength of $\lambda = 2100$ nm (within the tunable range of 1600–2500 nm), the corresponding angular frequency is

$$\omega = \frac{2\pi c}{\lambda} \approx 9.0 \times 10^{14}\ \text{rad}\,\text{s}^{-1}. \tag{11}$$

We take the work function of silver to be $\Phi = 4.6$ eV $= 7.37 \times 10^{-19}$ J. The Keldysh parameter is then given by

$$\gamma = \frac{\omega}{eE_0}\sqrt{2m_e\Phi}, \tag{12}$$

where e is the elementary charge and m_e is the electron mass. Inserting the above values yields

$$\gamma \approx 2.9. \tag{13}$$

Including near-field enhancement factors in the range $f \approx 5$–15 and accounting for variations in the silver work function ($\Phi \approx 4.2$–4.6 eV), the effective Keldysh parameter is reduced to

$$\gamma \approx 0.2\text{–}0.7. \tag{14}$$

Here, the lower bound corresponds to longer wavelengths, larger local field enhancement ($f \approx 15$), and a smaller work function ($\Phi \approx 4.2$ eV), while the upper bound corresponds to shorter wavelengths, smaller enhancement ($f \approx 5$), and a larger work function ($\Phi \approx 4.6$ eV). This range places the experiment firmly in the strong-field regime with $\gamma 1$, i.e. at the crossover between multiphoton and tunneling photoemission, depending on the exact local enhancement and surface conditions.

References

(1) Zhuo, X.; Zhu, X.; Li, Q.; Yang, Z.; Wang, J. *ACS Nano* **2015**, *9*, 7523–7535.

(2) Sun, Y.; Gates, B.; Mayers, B.; Xia, Y. *Nano Lett.* **2002**, *2*, 165–168.

(3) Mikkelsen, M. H.; Alerstam, E.; Johansson, P., et al. *Physical Review B* **2009**, *79*, 205413.

(4) Dorfmüller, J.; Vogelgesang, R.; Khunsin, W., et al. *Nano Letters* **2010**, *10*, 3596–3603.

(5) Mårsell, E. et al. *Nano Lett.* **2018**, *18*, 907–915.

(6) Mårsell, E.; Losquin, A.; Svärd, R.; Miranda, M.; Guo, C.; Harth, A.; Lorek, E.; Mauritsson, J.; Arnold, C. L.; Xu, H.; L'Huillier, A.; Mikkelsen, A. *Nano Lett.* **2015**, *15*, 6601–6608.

(7) Ltd., A. C. Ansys Lumerical FDTD Solutions, Version 2025 R1, Software, Vancouver, 2025.

(8) *Handbook of Optical Constants of Solids*; Palik, E. D., Ed.; Academic Press: New York, 1985.

(9) Anisimov, S. I.; Kapeliovich, B. L.; Perelman, T. L. *Zh. Eksp. Teor. Fiz.* **1974**, *66*, Sov. Phys. JETP 39, 375 (1974), 375–377.

(10) Madan, I.; Dias, E. J. C.; Gargiulo, S., et al. *ACS Nano* **2023**, *17*, 3657–3667.

(11) Lin, Z.; Zhigilei, L. V.; Celli, V. *Physical Review B* **2008**, *77*, 075133.

(12) Jackson, J. D., *Classical Electrodynamics*, 3rd ed.; John Wiley & Sons: 2021.

(13) García de Abajo, F. J. *Physical Review B* **1999**, *59*, 3095–3107.

(14) García de Abajo, F. J. *Physical Review B* **1999**, *60*, 6086–6102.
